# The present does not reflect the past: geographic mismatches between fossil and modern records are widespread across the tree of life

**DOI:** 10.1098/rspb.2026.1431

**Published:** 2026-10-21

**Authors:** Benjamin R. Shipley, Erin E. Saupe

**Affiliations:** Department of Earth Sciences, University of Oxford, Oxford, UK

**Keywords:** biogeography, dispersal, phylogenetics, species range, fossil record

## Abstract

The distribution of organisms across Earth provides insight into the origin and maintenance of biodiversity. Abundant fossil evidence suggests taxa have undergone substantial range changes over time. However, biogeographic theory and models often tend to assume that clades originated in the regions where we observe them today. Understanding the prevalence of geographically dynamic clades across the tree of life provides a way to evaluate these assumptions, thereby refining key evolutionary hypotheses. We provide a review of such range-shifting clades using a two-pronged approach. First, literature searches uncovered examples across most major multicellular groups (comprising 129 clades with mismatches between fossil and modern ranges). Concurrently, we used the well-sampled mammalian fossil record as a case study, finding that more than half of its terrestrial families (51%) had substantial (>1000 km) geographic mismatches. Geographically dynamic lineages are both common and widespread, but despite their ubiquity, fossil–modern mismatches have only recently been integrated into phylogenetic and biogeographic methods and no comprehensive overview of their advantages and challenges exists. We therefore review the current state of the field and discuss case studies where fossil evidence reshaped prevailing biogeographic theory. Biogeographers must take advantage of these rich fossil histories to better elucidate evolutionary theory across space and time.

## 1. Introduction

Our planet harbours an extraordinary diversity of life that is scattered unequally across geographic space. For at least the last few million years, lower latitudes, coral reefs and mountain ranges have maintained particularly high levels of diversity across many taxonomic groups [1–4]. Spatial patterns of diversification, extinction, dispersal and evolution combine to modify and maintain biodiversity, and how these patterns arose and interact with each other are the field-defining question of biogeography [5,6]. Species distributions are often crucial in understanding these ecological and evolutionary processes. Distributional patterns can explain where and how clades originated [7–11], why rare species persist in certain areas [12,13] and how the climate and geography of the Earth have changed through time [14–16]. Where species live today, however, is often not where they lived in the past [17,18]. The fossil record tells us that the distributions of many taxa have changed dramatically throughout Earth's history ( figure 1) [17–22]. For example, crocodiles are today restricted to lower latitudes, but the fossil record shows that they lived in the high Arctic during the Palaeogene [23]. More recently, fossils dating to the Pleistocene reveal that members of the sub-Saharan hippopotamus and giraffe families once lived across Eurasia [24–27].

**Figure 1.**
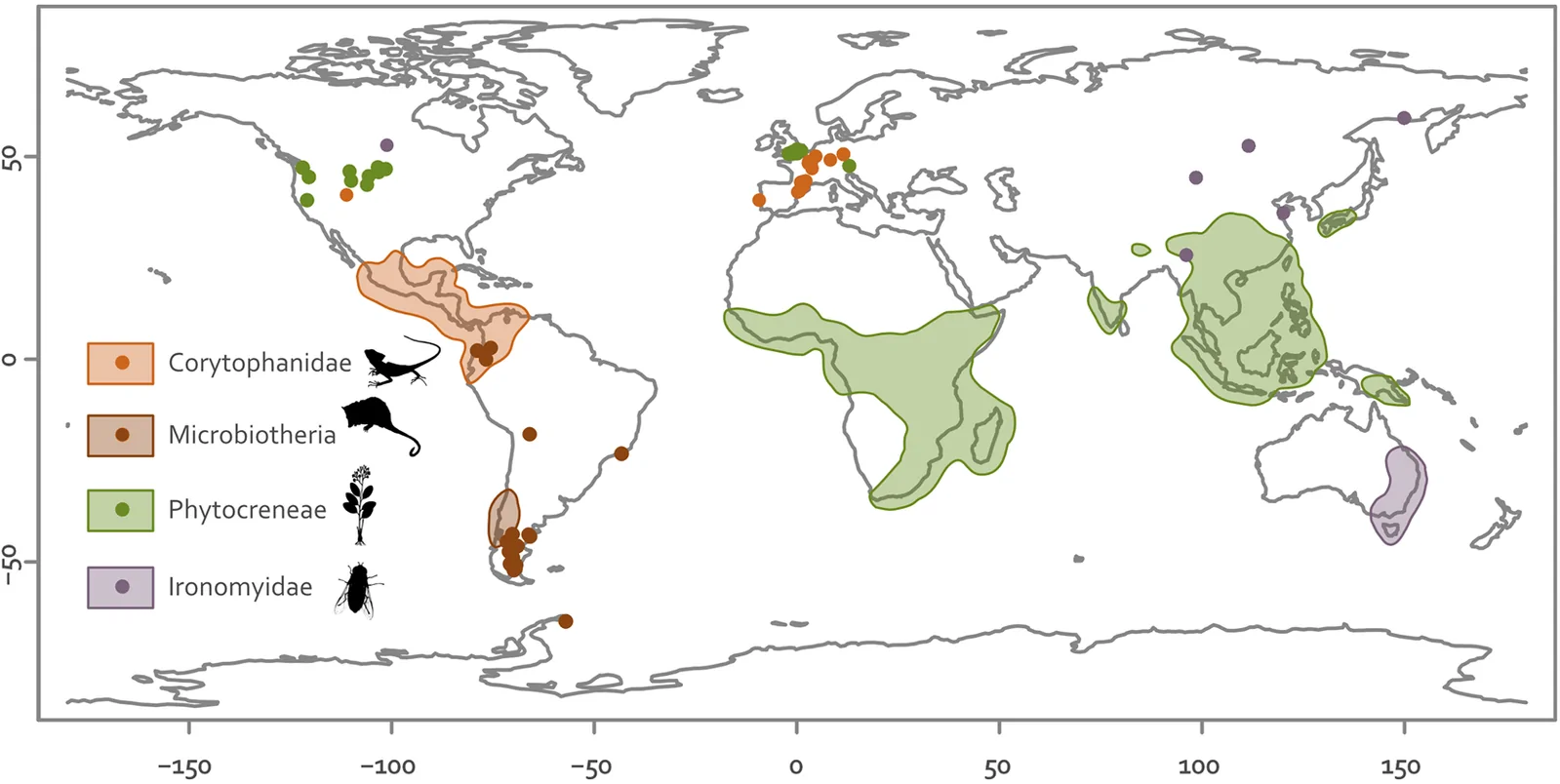
Four examples of taxa with discrepancies between the modern record (shaded areas) and fossil record (points; not palaeo-rotated). The four clades were chosen because their modern geographic distributions do not overlap. Icons are from Phylopic: Microbiotheria (*Dromiciops gliroides* by Sarah Werning. CC BY 3.0), Corytophanidae (*Laemanctus serratus* by Jose Carlos Arenas-Monroy. CC0 1.0), Phytocreneae (*Icacina oliviformis* by Guillaume Dera. CC0 1.0) and Ironomyidae (*Syrphidae* by Sean McCann. CC0 1.0).

It is clear that taxa with different fossil and modern distributions can illuminate the mechanisms driving the unequal distribution of life on Earth [28–34]. These taxa have supported and refuted classic hypotheses that explain patterns in biodiversity and evolution [35], such as the relative importance of vicariance and dispersal in structuring modern biodiversity [17,34,36,37], and have suggested that some modern distributions are relicts of much broader distributions (e.g. the Boreotropical hypothesis [38–41]). For decades, researchers have used spatial analyses to describe range dynamics in both modern and fossil lineages [42,43]. However, despite abundant evidence of changes in distribution through time across the tree of life, and theoretical work that calls for a dynamic understanding of species distributions and incorporation of the fossil record [17,31,44], many current methods and paradigms in ecology and evolution still rely on the assumption that taxa must have lived in the past where they occur today. For example, ancestral range reconstructions, even ones that incorporate the fossil record, assume that the range of a taxon does not change from origination to observation [45–48]. Similarly, estimates of speciation, extinction and diversity through time often rely on snapshots through time, commonly the present day, which may not reflect the continuous, dynamic nature of distributional shifts [49,50].

Part of the disconnect between the theoretical value of incorporating fossils into biogeographic inference and their practical application may arise from limited information on how frequently continental-scale distribution shifts occur and from insufficient communication of these patterns. The fossil record is sparse and taxonomically biased and identification of fossils requires specialist knowledge that often does not overlap with the methods used to interrogate large-scale spatial hypotheses. Moreover, most literature documenting extra-limital occurrences tends to focus on relatively fine taxonomic scales (e.g. [19,51]), such that researchers specializing in the modern biogeography of a clade without a well-known fossil record may not intrinsically consider incorporating fossils in their analyses. Even if a clade does have a well-described fossil record, in some cases the mismatch in tools, techniques, timescales and communication between neontological and palaeontological research hinders the use of fossils in biogeographic inference [52].

To illuminate complex biogeographic patterns and processes, we must account for distributional changes through time and the prevalence of extant clades with different fossil and modern distributions. In this work, we provide a cross-taxon review of clades that have different fossil and modern distributions, using a two-pronged approach to interrogate the prevalence of these clades. First, we examine the breadth of these disjunct taxa across the tree of life, reviewing the literature for extant clades that occupy substantially different distributions than they did in the past (figure 1). Second, we use the well-sampled fossil record of terrestrial mammals as a case study to show how prevalent these disjunct clades are within smaller taxonomic groups. Finally, we explore the implications of the widespread mismatches between fossil and modern ranges for fundamental theory in biogeography, palaeontology and macroevolution, and highlight recently developed tools that account for these mismatches. Through this work, we emphasize the dynamic nature and history of clades across the surface of the Earth and caution against over-interpreting historical narratives derived solely from extant biogeographic patterns.

## 2. How common are mismatches between fossil and modern distributions?

### (a) Literature review: the taxonomic breadth of geographic discrepancies

Between 15 June and 15 September 2024 and from 26 November to 15 December 2025, we conducted an extensive review of clades with different fossil and modern distributions (supplementary material, appendix SI). When a potential candidate clade was identified through the literature, we gathered occurrence data from the Palaeobiology Database (PBDB) [53] and the Global Biodiversity Information Facility (GBIF) [54], and an additional review of the literature. We then cleaned and filtered the fossil and modern occurrences, removing occurrences with unknown provenance and high spatial and temporal uncertainty (supplementary material). We defined a clade as having undergone distributional changes if the available fossil occurrences of the clade fell outside of the combined distribution of all extant species. We standardized the taxonomy of the clades based on the literature (supplementary material, appendix S1). The literature search involved 523 published works, uncovering 132 clades across the tree of life with geographic mismatches between the fossil and modern records (supplementary material, appendix S1). These clades varied across taxonomic levels from genus to order, although most were at the family level or lower (supplementary material, appendix S1). Mammals, insects and birds were the best-represented taxa in our cross-taxon review, with 35, 28 and 19 clades, respectively (figures 1 and 2).

**Figure 2.**
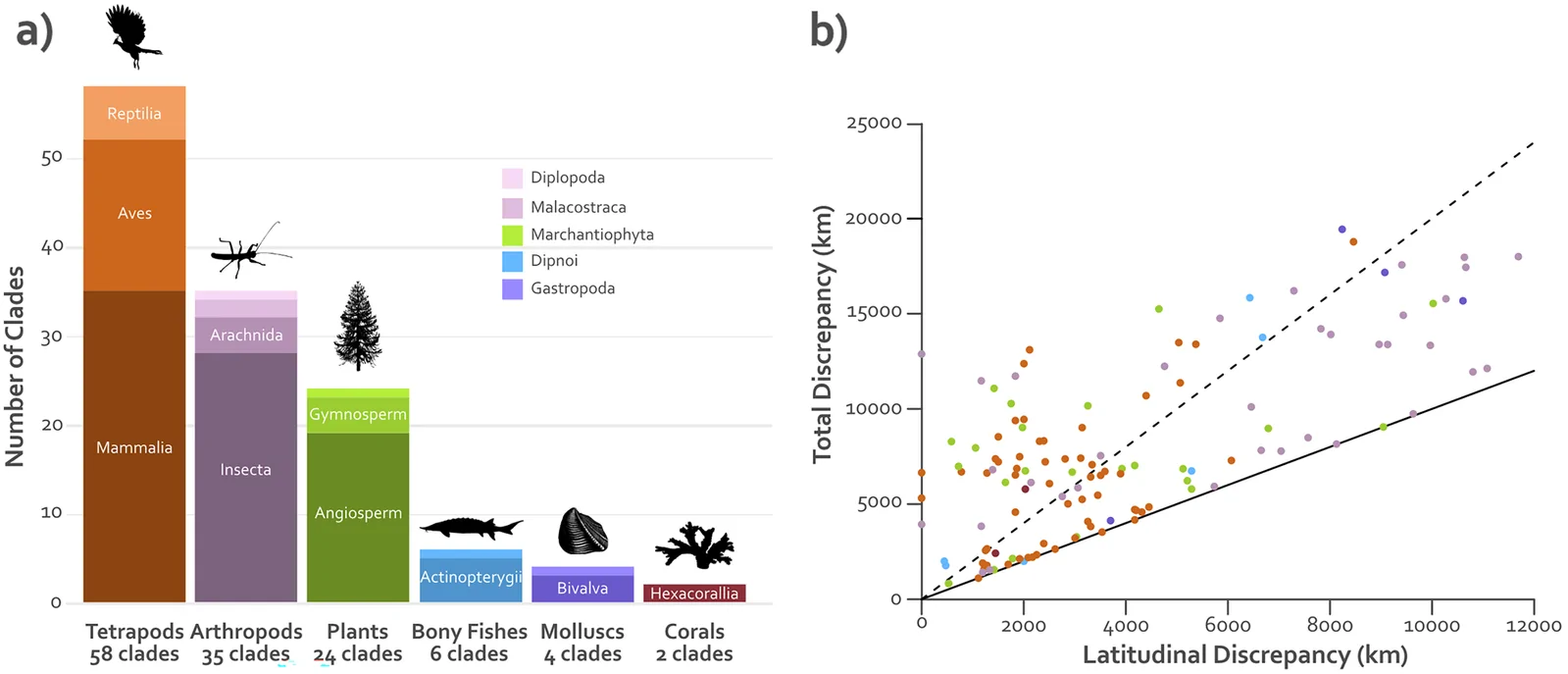
(a) The number of clades examined with substantial total (>1000 km) or latitudinal (>500 km) discrepancies between their fossil and modern records (*n* = 129), and (b) the relationship between total and latitudinal discrepancies in each of those 129 clades. The solid line indicates where total = latitudinal discrepancies (i.e. exclusively latitudinal discrepancies) and the dashed line indicates discrepancies that are equally large latitudinally and longitudinally. Icon attributions: tetrapods (*Opisthocomus hoazin* by Warren H (photography)), T. Michael Keesey (vectorization. CC BY 3.0), arthropods (*Mantophasma zephyrum* by Darren J. Parker. CC0 1.0), plants (*Araucaria araucana* by Mason Mcnair. CC0 1.0), bony fishes (*Acipenser oxyrhynchus* by Duane Raver/Usfws. Public Domain mark 1.0), molluscs (*Trigonia*, adapted from [55]) and corals (*Acropora florida* by Guillaume Dera. CC0 1.0).

To determine whether the mismatches suggest actual distributional changes or are instead owing to plate tectonics, we palaeo-rotated the fossil occurrences (supplementary material, appendix SII) and measured the geographic discrepancies between the fossil and modern occurrences in two ways. First, we constructed convex hulls around the modern occurrences and measured the distance from the palaeo-rotated fossil occurrences to the convex hull (hereafter, total discrepancy). If the modern occurrences crossed the anti-meridian (e.g. pan-Pacific species), we rotated both the fossil and modern occurrences such that the anti-meridian crossed through the Atlantic Ocean instead. Second, we calculated how far the latitudinal ranges of the palaeo-rotated fossil data extend beyond modern latitudinal ranges (hereafter, latitudinal discrepancy). Whereas ‘total discrepancy’ is measured from the convex hull around the modern occurrences, ‘latitudinal discrepancy’ is measured from the maximum or minimum latitude of the modern occurrences. Therefore, the two metrics provide different information on how the distribution of the clade has changed through time. Because of increased uncertainty in estimating palaeolongitude from tectonic models [56], the latitudinal discrepancies provide a more robust, albeit conservative, estimate of the extent of these distributional dynamics.

We limited clades to those with total discrepancies >1000 km or latitudinal discrepancies >500 km to account for uncertainty in the palaeo-coordinate models and the time averaging of the fossils (supplementary material, appendix SII), leaving 129 clades (figure 2a; supplementary material, tables S1 and S2). The average total discrepancy of the 129 clades was 7878 km (s.d. = 4652 km). The gastropod genus *Campanile* had the largest total discrepancy of 19 425 km, with a cosmopolitan fossil record and a modern range restricted to southwestern Australia. The scorpionfly family Eomeropidae (Mecoptera) had the largest latitudinal discrepancy of 11 688 km, with a modern range in Patagonia and fossil occurrences in northern Europe.

As expected, the total and latitudinal discrepancies we observed across the 129 clades were strongly correlated (Spearman's *ρ* = 0.607); taxa with fossil occurrences farther outside the convex hull of their modern distribution also tended to have fossil occurrences in different latitudes from their modern distribution. However, the degree to which the distribution of each clade experienced longitudinal versus latitudinal changes varied substantially (figure 2b). The number of clades with greater longitudinal (east–west) discrepancies and greater latitudinal (north–south) discrepancies were approximately equal (58 versus 71 clades, respectively, figure 2b). Four clades had predominantly longitudinal rather than latitudinal discrepancies (latitudinal discrepancy < 100 km; supplementary material, table S2). For example, hummingbirds (Trochilidae) today live only in North and South America, but the hummingbird fossil record includes European occurrences during the Oligocene [57]. Similarly, nine clades exhibited latitudinal discrepancies without longitudinal ones, including the sub-Saharan family Bucorvidae (hornbills), which have known fossils in northern Africa and the Mediterranean.

Although our analyses span several levels of taxonomic resolution (from genus to order), the taxonomic level of the clade did not influence the magnitude of the discrepancies (Kruskal–Wallis test, *n* = 129, total discrepancies; d.f. = 4, *χ*^2^ = 7.61, *p* = 0.106, latitudinal discrepancies; *χ*^2^ = 0.734, *p* = 0.947). Many of the clades were species-depauperate, containing only a few extant species within a larger clade; of the 129 clades identified, 19 had only one extant species within their families and an additional eight clades had only two extant species.

### (b) Unequal distributional dynamics: the effect of sampling bias

In addition to latitudinal and longitudinal discrepancies, we examined changes in geographical realm for these 129 clades. First, we divided the world into eight modern geographic realms based on the scheme provided by [58] and characterized the realms in which each clade currently lives based on their modern occurrence data (figure 3). We then compared the current geographic realms of the modern occurrences to those of the fossil occurrences. Crucially, we intend this analysis to show only changes in the geographic locations of the fossil and modern occurrences, not their environmental conditions. For example, during the Eocene, the Palaearctic mid-latitudes had similar environmental conditions to the tropics today, so a modern-day tropical taxon with Eocene-age fossils in the Palaearctic would (likely) not have changed its environmental niche, but would still have undergone a realm-scale shift in its geographic distribution. Most of the clades we reviewed (*n* = 105 out of 129) had fossil records in different geographic realms from their modern ranges (figure 3). The clades that changed their distributions across realms did so unequally: fossil records from the Nearctic and Palaearctic realms were much more likely to represent modern Gondwanan taxa (Australasian, Indo-malayan, Afrotropical and Neotropical realms) than vice versa (figure 3).

**Figure 3.**
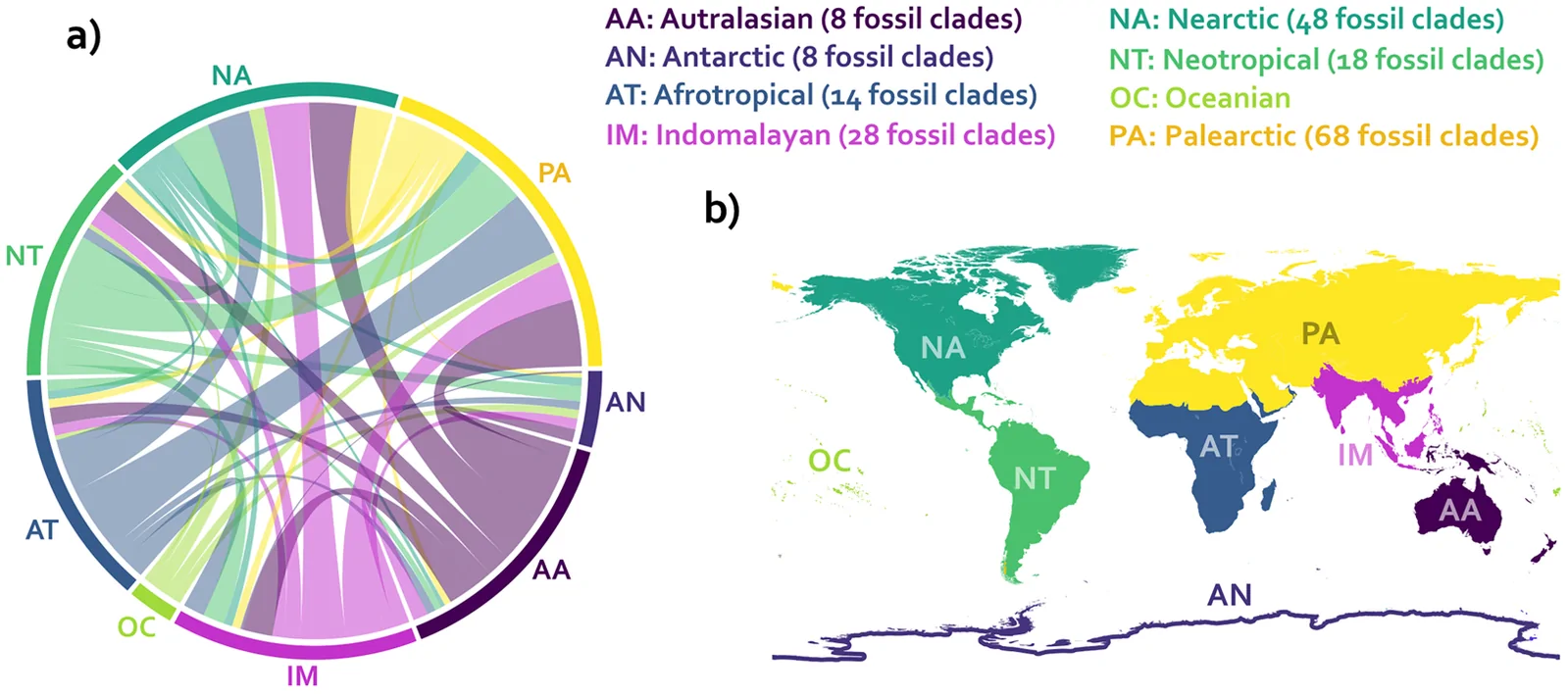
(a) Chord diagram showing the 105 clades we reviewed with fossil records in different geographic realms from their modern occurrences. The colour of the link indicates the realm in which the clade currently resides. Each clade can have multiple links if it currently resides in multiple realms or if it has fossil occurrences across multiple realms. For example, elephants (family Elephantidae) are today found only in the AT and IM realms, but they have fossil records in the PA and NA realms, so four separate links are created: AT-PA (blue), AT-NA (blue), IM-PA (fuchsia) and IM-NA (fuchsia). (b) A map of the eight biogeographic realms used in this analysis, based on [58]. The number of fossil clades belonging to each realm is listed above the map. The links show changes in geography not environment or habitat; for example, a link between PA and IM does not imply a change in environmental conditions, only geographic shifts. A modern-day tropical taxon found in the Palaearctic of the Eocene may have existed in similar environmental conditions to the tropics today.

The results of any biogeographic analysis using the fossil record, and especially studies examining palaeontological distributions, depend substantially on Lagerstätten (exceptionally well-preserved or fossiliferous formations), such as the Eocene amber deposits in northeastern Europe and Myanmar, which represent remnants of rain forests that once covered much of Laurasia [59], and the Monte Bolca assemblage of reef fish [60]. Lagerstätten and fossiliferous regions in general are biased towards northern latitudes, with relatively few fossil localities in the tropics or Southern Hemisphere [61–63]. Differences in available funding and palaeontological resources across countries exacerbate this unequal distribution [64], as do the inherently incomplete nature of the PBDB and GBIF (from which many of the occurrences were derived). In particular, because we only used fossil occurrences associated with published literature or those that had a written history of identification, dating, location matching and provenance (supplementary material, appendix S1) regions with a greater body of literature are likely to be overrepresented in this study. Therefore, it is unsurprising that we observe many Gondwanan extant species with Northern Hemisphere fossil occurrences, but few Northern Hemisphere extant species with Gondwanan fossil occurrences (figure 3). Expanding funding and sampling initiatives in regions historically neglected by palaeontologists is, therefore, critical for understanding biogeographical patterns through time. In addition to sampling biases, differences in preservation potential across fossil localities can introduce bias in biogeographic studies. For example, amber deposits preserve small or soft-bodied taxa better than sediments [65–67]. These biases and incomplete fossil sampling limit our ability to distinguish range shifts from range contractions (supplementary material, appendix SIII). Despite these spatial biases in the fossil record, the discrepancies we observed in this study are likely real (and, in fact, underestimate the actual discrepancies experienced by the clades), because we have good knowledge of modern distributions for most clades. The clades and fossils examined in this study therefore provide positive evidence of past distributions that are dramatically different from present-day ranges, albeit dependent on taxonomy. Although fossils provide real evidence of species occurrence, they are not without uncertainty or error. In particular, identification of fossils and their taxonomic placement has remained highly contentious for over 200 years [68,69]. For example, a Chinese fossil was identified as a member of a clade of North American pitcher plants [70], suggesting a dramatic geographic discrepancy. However, 10 years later, it was redescribed as an insect gall [71]. Some of the fossils used here to characterize geographic discrepancies might similarly be reclassified as completely different taxa in the future.

### (c) Case study: the prevalence of geographic discrepancies within a clade

To supplement our literature review and determine the prevalence of clades with discrepancies between their fossil and modern distributions within a broader taxonomic group, we examined every modern terrestrial mammal family with a known fossil record (*n* = 127; figure 4). At relatively broad taxonomic resolutions (i.e. families within a class), clades with substantial geographic discrepancies appear to be common. The majority of extant terrestrial mammal families (class Mammalia) with a known fossil record in the PBDB (*n* = 127) had geographic discrepancies (median total discrepancy: 1082 km; median latitudinal discrepancy: 362 km; figure 4). Out of these 127 families, 65 (51.1%) had total discrepancies of >1000 km from their expert-generated modern ranges, with four families having more than a 12 000 km difference between their fossil and modern records (maximum = 13 134 km; figure 4a). When considering latitudinal changes, slightly fewer species showed substantial discrepancies, with only 57 families (44.9%) that had fossil distributions more than 500 km outside of their modern latitudinal ranges (figure 4c).

**Figure 4.**
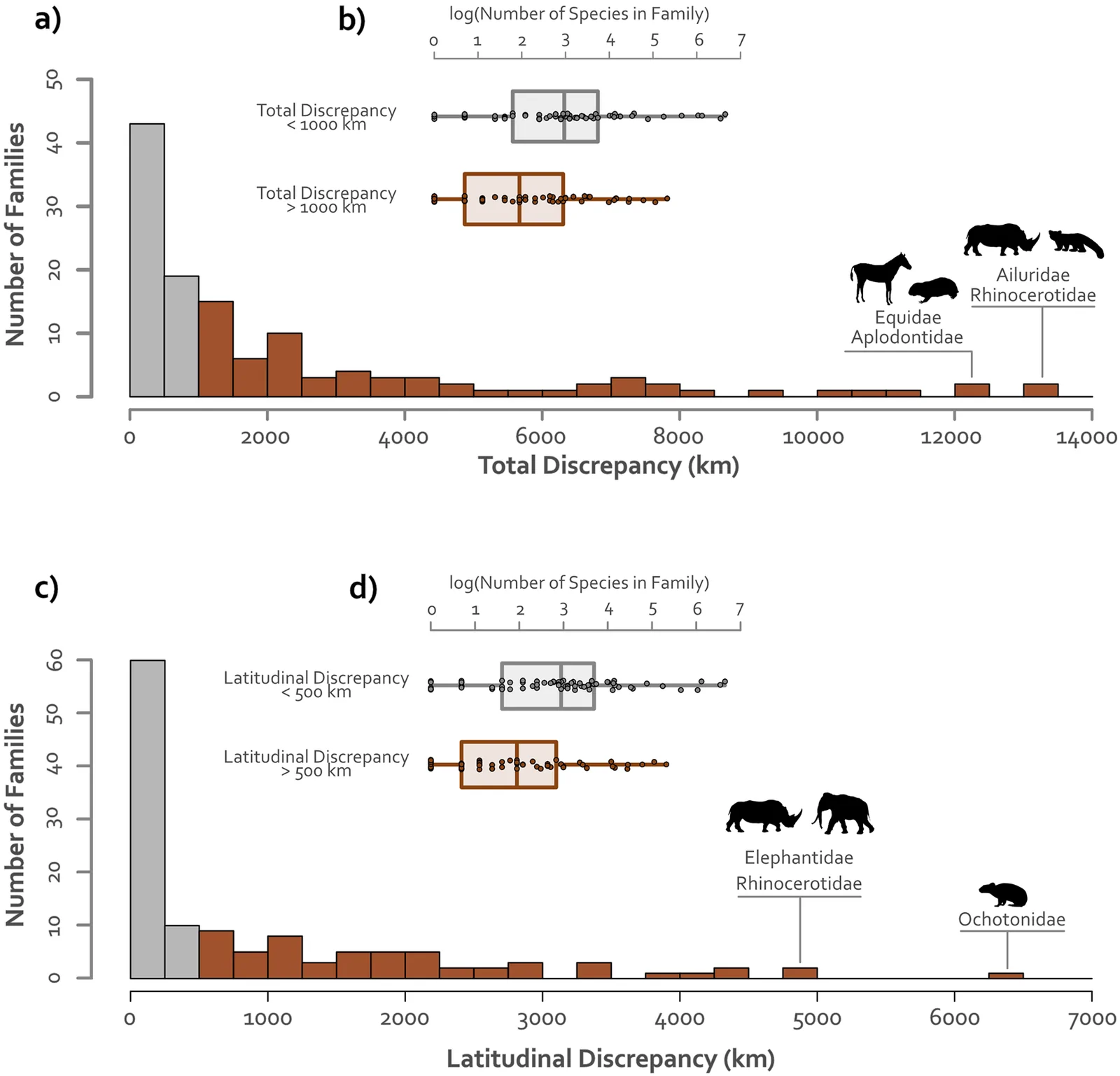
Analysis of the 127 mammal families with modern and fossil records, comparing those with geographic discrepancies to those without. Top row: total discrepancies. (a) Frequency distribution of total discrepancies, with discrepancies >1000 km coloured brown. (b) Box plots comparing species richness of the mammal families with discrepancies <1000 km (top and grey box plot) to those with discrepancies >1000 km (bottom and brown box plot). Bottom row: latitudinal discrepancies. (c) Frequency distribution of latitudinal discrepancies, with discrepancies >500 km coloured brown. (d) Box plots comparing species richness of the mammal families with discrepancies <500 km (top and grey box plot) to those with discrepancies >500 km (bottom and brown box plot). Families with substantial differences between their fossil and modern records have significantly fewer species. Icon attribution: Equidae (*Pliohippus* by Zimices, Julián Bayona. CC BY 3.0), Aplodontidae (*Aplodontia rufa* by Diderot. CC BY 3.0), Ailuridae (*Ailurus fulgens* by Xavier A. Jenkins. CC0 1.0), Rhinocerotidae (*Ceratotherium simum* by Christophe Mallet. CC0 1.0), Elephantidae (*Palaeoloxodon antiquus* by Steven Traver. CC0 1.0) and Ochotonidae (*Prolagus sardus* by Steven Traver. CC0 1.0).

The relationship between total discrepancy and latitudinal discrepancy in mammal families was stronger than in the clades uncovered in our literature review (Spearman's *ρ* = 0.838); however, we found a high variance in the direction of the geographic discrepancies for mammal families. Of the 68 mammal families that had substantial discrepancies (total > 1000 km and latitudinal > 500 km), 34 had greater latitudinal discrepancies than longitudinal ones and 34 had greater longitudinal than latitudinal discrepancies. Six families underwent longitudinal distributional changes, compared with seven families that underwent predominantly latitudinal distributional changes. Overall, geographically dynamic mammal families comprised fewer extant species than the families that did not have latitude discrepancies (figure 4b,d; Wilcoxon rank sum tests: *n* = 127, total discrepancy *W* = 2703, CI95 = [–3.0, –17.0] and latitudinal discrepancy *W* = 2657, CI95 = [–3.0, –16.0]).

## 3. Implications and outlook

### (a) Summary

The results of our cross-taxon literature review suggest that substantial, continental-scale differences in the distribution of clades between the past and the present occur across the tree of life (figure 2a), and our analysis of every mammal family showed that they are surprisingly common (figure 4).

These estimates of distributional change are certainly conservative for many reasons. First, our literature review was not exhaustive; in our mammalian case study alone, several families had geographic discrepancies that we did not encounter during our literature search. These families represent taxa in which the extra-limital fossils did not warrant discussion in the identifying literature, either because: (i) the distributional change has been well known for decades (e.g. Viverridae [72]), (ii) the fossil taxon was recently reassigned to another family or its taxonomic affinity is uncertain (e.g. Pitheciidae [73]), or (iii) the authors may not have considered the range expansion of the clade sufficiently noteworthy to remark upon. Furthermore, our strict filtering requirements for fossil occurrences likely lead to conservative estimates of where the clades lived in the past. The preliminary results discussed here are therefore only a subset of clades with geographic discrepancies, which very likely make up a considerably larger portion of all extant biodiversity on Earth. Even for those clades in which we do report discrepancies, the relative under-sampling of the fossil record compared to the modern record means that the discrepancies are only minimum values; it is likely that some taxa lived even farther away from their modern records than we were able to detect (supplementary material, appendix SIII).

The spatial and taxonomic bias in the PBDB [74], GBIF and the fossil record itself [61–63] additionally obscures large-scale shifts in geographical range, especially in groups that are understudied in the modern or those with poor taphonomy. The PBDB is based on published literature, which ignores unpublished data housed in public and private museums [75]. However, imprecise data entry and outdated taxonomy lead to errors of commission [76]. We attempted to mitigate these issues by (i) supplementing the PBDB observations with those from our literature review, from GBIF, and, for mammals, from the New and Old Worlds database [77], and (ii) comprehensively re-examining the taxonomic affiliations of the observations (supplementary material, table S1). However, as with all analyses, involving large biodiversity databases, the patterns observed here are highly influenced by the quality of the data we used.

Taxa may exhibit geographical discrepancies for many reasons. First, they may track environmental dynamics, changing their distributions in response to the availability of climates within their ecological niches [78,79]. The changes in distribution for some taxa, such as land snails [80], turtles [22] and foraminifera [81], are thought to be strongly constrained by climatic factors. In times of substantial community turnover, invasion and extinction, however, taxa may sometimes undergo niche change [82–85]. Although niche models in the fossil record suggest niche stability is more common than niche change at least within lineages on short timescales [86,87], the effect of niche stability or change on geographic range shifts across evolutionary timescales is still unclear [87].

Extinction is a second proposed mechanism for the substantial distributional discrepancies between the fossil and modern locations in some taxa. Extinctions eliminate taxa from areas where they once lived. These extinctions may result from biotic or abiotic changes and could select against certain traits or environments [88]. Consequently, a once-speciose clade with only a few modern survivors may have a much smaller modern range than in the past. For example, the basal lizard-like clade Rhynchocephalia was biodiverse and widespread throughout much of the Mesozoic, but rhynchocephalians went extinct in most parts of the world by the early Cenozoic, and the clade is now represented by only one species endemic to New Zealand, the tuatara (*Sphenodon punctatus*) [89,90].

Finally, older lineages are more likely to have experienced both large-scale climate changes and extinction events relative to younger clades, and therefore may be more likely to show substantial distributional changes. The age of a clade itself may also influence its tendency to undergo these changes. Diversification rates within a clade tend to decline through time, perhaps because of small initial populations, isolated and small range sizes and reduced genetic diversity of a founder population immediately after a speciation event [91]; this means an older clade may not have the intraspecific variation necessary to diversify into previously unoccupied morphological or niche space [91], limiting its ability to expand its geographic distribution or to rebound after a range contraction [92]. Previous literature has demonstrated that the fossil record is essential for accurately identifying which of these proposed mechanisms caused the discrepancy [17,28–31,33,44,93–95]. However, our results emphasize the scope of this problem and the extent to which the fossil record can change biogeographical models.

In this work, we found approximately equal latitudinal and longitudinal discrepancies (e.g. figure 2b) for both our cross-taxon review and our case study of mammal families, suggesting that changes in clade distributions over long timescales may not be predominantly north–south or east–west. Given the propensity of modern tropical species to have fossil records at higher latitudes (figure 3), this result is somewhat surprising. Latitudinal shifts in clade distributions are often hypothesized to occur owing to climatic fluctuations [22,86], whereas longitudinal shifts may stem from patterns of dispersal (e.g. over land bridges), regional extinction and tectonics [39,57,96]. Our findings suggest that both niche and dispersal dynamics act in tandem within a clade to constrain and drive changes in its distribution over evolutionary time. Furthermore, the taxonomic level of the clade did not substantially affect how distant the fossil occurrences were from the modern locations, possibly because the clades with the largest discrepancies had few species, regardless of their taxonomic level. The relative dearth of extant species in clades with substantial discrepancies suggests that patterns of extinction may drive geographic mismatches between the fossil and modern records. If enough taxa within a clade go extinct, geographic discrepancies can likely occur through random chance. However, extinctions often select against certain traits and environments, particularly in regions with variable environments [88], which may exacerbate these discrepancies.

### (b) Biogeographical implications of geographic discrepancies

The prevalent and substantial changes in distribution we observed across life on Earth have far-reaching implications. First, they challenge our assumptions on where taxa originated and lineages diverged. These assumptions are often based on parsimony. For example, the mammal families Anomaluridae and Pedetidae are closely related and are both endemic to sub-Saharan Africa, suggesting they originated in the region. However, the fossil records of both families reveal they likely diverged somewhere in Asia and later dispersed to Africa [97]. Taxa with ‘Gondwanan distributions’ (i.e. Neotropical, Afrotropical and Australian distributions) are often assumed to have diverged when Gondwana broke up roughly 180–140 Ma, but fossils of many putative ‘Gondwanan’ clades have been found in the Northern Hemisphere (figure 3), complicating these assumptions [19,98].

These large-scale changes in distribution demonstrate that our inferred mechanisms of diversification and biogeographic dynamics in many clades may be incorrect without considering the age and distribution of the fossil record [30,47,93–95,99]. For example, phylogenies that only use extant taxa suggest that the radiation of lungfish (Dipnoi) occurred either well before [100] or well after the fragmentation of Gondwana [101], removing continental vicariance as a mechanism driving the diversification of the clade. However, a study integrating the fossil record into these phylogenies showed that lungfish lineages may have diversified concurrently with (and therefore potentially because of) Gondwanan breakup [37]. Similarly, new fossils of early reef fish in Mexico have overturned earlier biogeographic reconstructions describing the Indo-Australian archipelago as the primary hotspot of ancestral lineages [102]. Incorporating fossils into these models is not a panacea—issues of non-identifiability (the inability to distinguish between multiple biogeographic hypotheses) still plague molecular phylogenies even when some fossils are included [93,103,104]. However, the strong temporal and spatial constraints provided by the fossil record can substantially mitigate these issues [93,105,106].

The mismatch between the fossil and modern records could also influence our interpretations of modern-day patterns and inferred processes, such as the latitudinal diversity gradient [103]. The same patterns could arise from multiple different mechanisms, and incorporating extra-limital fossil occurrences can help to parse the drivers of these patterns. For example, mammalian carnivores demonstrate a traditional latitudinal richness gradient (higher richness in the tropics), which has been proposed to result from the well-known ‘tropics as a museum’ hypothesis, which suggests that the clade originated in the lower latitudes [49,107]. However, fossil evidence suggests that carnivores diversified in the northern temperate latitudes before dispersing south [108]. Without consideration of the fossil record, our assumptions on the proposed mechanisms generating biogeographic patterns, such as higher richness at low latitudes, may be inaccurate, as speciation and extinction dynamics in the past will be difficult to infer. Thus, sole reliance on modern distributions may risk errors when inferring rates and patterns of speciation, extinction and diversification and their associated drivers [98,103].

### (c) Tools for incorporating large-scale distributional changes into biogeographic hypotheses

Recently, some tools and methods have explicitly accounted for distributional changes when forming biogeographic hypotheses. Many of these approaches are empirical, using the actual distributions of the fossils and their ages to improve our understanding of biogeography, such as quantifying ‘present natural’ ranges of extant mammalian species [109]. These ranges use historical and palaeontological records to hypothesize current species’ distributions in the absence of human effects, and they can be used to evaluate how robust current biogeographic theory is to the intense changes caused by humans throughout the Holocene. On longer timescales, directly incorporating fossils into phylogenetic methods can allow for improved estimates of diversification and extinction across both space and time [29,34,47,93,110]. For example, many stochastic birth–death models (reviewed in [110]) allow for fossil nodes, which mitigates the bias caused by examining only extant taxa and allows for additional nuance in exploring diversification rates through time and across a phylogeny. With the development of high-resolution climate models across the Phanerozoic (e.g. [111]), incorporating fossils into ecological niche models can also provide new insights into biogeographic patterns and niche evolution [86].

Despite these methodological advances, characterizing distributional changes using empirical data will likely remain challenging for some regions and clades owing to the sparse nature of the fossil record. Although we can potentially estimate the relative rank-order range sizes of taxa across time by simplifying geographic ranges into latitudinal extents, convex hulls and great circle distances [112], the fossil record can be patchy and therefore range dynamics are often unclear (supplementary material, appendix SIII). In the absence of empirical information, simulations and modelling approaches can interrogate hypothesized geographical dynamics under certain methodological assumptions and limitations. For example, the dispersal–extinction–cladogenesis (DEC) model used in ancestral state reconstruction [9] integrates the locations and ages of both modern and fossil occurrences to infer large-scale patterns of diversification and to test biogeographic hypotheses [34,40]. The BioGeoBEARS software package [113] expands on DEC models incorporating jump and oceanic dispersal [114] to refine biogeographic hypotheses. However, these models have well-known methodological limitations [93,115,116], such as assuming no lineage extinction. More recently, the dispersal–extinction–sampling model can infer rates of dispersal, speciation and extinction concurrently with estimates of range evolution [117]. If the assumptions of these models are met, incorporation of the fossil record can sometimes lead to more robust estimates of ancestral ranges, especially in older nodes [93,95]. Finally, niche-based virtual species simulations can be used to examine patterns of speciation and extinction and diversity dynamics through time, given the assumptions of niche stability [86,118,119] or predictable niche evolution [120–122]. Although all of these tools have their limitations and none provide ironclad evidence in the absence of known observations, they allow researchers to interrogate existing biogeographical hypotheses and to develop their own in concert with our constantly improving fossil record.

### (d) Conservation implications of widespread geographic mismatches

Understanding past geographical discrepancies can assist in the discovery and conservation of extant species. For example, the extant members of the spider ant genus *Leptomyrmex* were historically thought to be endemic to the rainforests of Australia, New Guinea and New Caledonia, despite a cosmopolitan fossil distribution [123,124]. However, in 2016, a new species of *Leptomyrmex* was discovered in Brazil, in a lineage thought extinct [123]. The fossil record can help to predict and find ‘Lazarus taxa’ such as these, especially in cryptic and understudied clades living in inaccessible regions. Incorporating fossil records into analyses of niche evolution and tracking can help to improve conservation of vulnerable species in the context of our current changing climate and could highlight new regions where taxa might survive [125–127], particularly if the modern realized niches of many clades do not reflect the realized niche of the clade in the past.

## 4. What are the next avenues to explore?

Continental-scale shifts in geographic distributions are both widespread across the tree of life and prevalent within single groups, and these distributional changes substantially influence hypotheses on the origin and maintenance of biogeographic patterns. We often do not know the specific mechanisms leading to changes in the distributions of these clades and many hypotheses—from niche dynamics to extinctions to clade age—have arisen to explain distributional shifts on a case-by-case basis. No one mechanism is likely to govern all of these distributional shifts; however, we have not yet disentangled the effects of climate change and extinctions from the intrinsic effects of age on mismatches between fossil and modern records, and the combined influence of speciation rate, niche evolution and taxon age on geographic range expansions largely remains untested. More work is therefore needed to tease apart the effects of niche change, extinction and tectonics on these continental-scale range dynamics. In particular, future research could continue to probe the effects of taxonomic scale and extinctions on geographic mismatches between the fossil and modern records, either empirically or using virtual simulations through time (see [29,51,86,87,128] for some examples). Rapid climate change, sea level rise, biological invasions, and anthropogenic effects have all caused large-scale redistributions of life throughout Earth's geologic history [129–131]. These changes leave lasting echoes in the fabric of life, contributing to the patterns we observe today. Accounting for distributional changes is therefore a substantial and necessary step to refining biogeographic inquiry. Fossils remain the only falsifiable evidence of these changes through time, allowing us to re-examine and reconstruct these biogeographical patterns to improve hypotheses about the history of life on Earth.

## Supplementary Material



## Data Availability

The data and code used to conduct the analyses described in this article are publicly accessible on FigShare at [132]. Supplementary material is available online [133].
