# Supplementary material for: The present does not reflect the past: geographic mismatches between fossil and modern records are widespread across the tree of life
Source: Proc Biol Sci. 2026 Oct 21;293(2081):20261431. doi: 10.1098/rspb.2026.1431 (PMC13682928; doi:10.1098/rspb.2026.1431)
Supplement: Supplementary file 1 [file rspb.2026.1431.zip › rspb.2026.1431/rspb20261431_si_001.docx]

**Supplementary Information:**

This file includes the following supplementary information for *The present does not reflect the past: Geographic mismatches between fossil and modern records are widespread across the tree of life:*

SI Methods

SI Appendices I to III

Table S1

Figures S1-S2

Legend for Table S2

Supplementary Reference List

**SI Methods:**

*Literature Search:*

Between 15 June and 15 September 2024, we searched Google Scholar for literature relating to the terms “disjunct distributions”, “relict species”, “extralimital fossil”, “living fossil”, “boreotropical”, “range contractions”, and combinations and subsets of those phrases. We used the snowball method, exhaustively searching both the literature cited and the citing literature for each article that described a clade with different fossil and modern distributions. This method is substantially more likely to return little-known literature than simply examining the results of the first-order literature search (1).

The clades we analysed varied from genera to orders. Most of the clades were at the family level or lower, with some exceptions. First, we included orders or suborders with only one extant family and at least one stem taxon. These include Coleorrhyncha (moss bugs), Coliiformes (mousebirds), Leptosomiformes (cuckoo-roller), Macroscelidea (elephant shrews), Microbiotheria (monito del monte), Musophagiformes (turacos), Opilioacarida (mites), Opisthocomiformes (hoatzin), Podargiformes (frogmouths), Rynchocephalia (tuatara), and Tubulidentata (aardvark). Additionally, we included Ceratodontiformes (lungfish), which contains three extant families; Coracii (roller and ground-roller), containing two; Crocodilia (crocodiles), containing three; Cycadales (cycads), containing two; Phreatoicidea (isopods), containing six; and Psittaciformes, containing four. See Supplementary Appendix I and Table S1 for a full list of clades. All subsequent data management and analyses were conducted in R v. 4.4 (2), using the “terra” (v. 1.8, 3), “fossilbrush” (v. 1.0, 4), “palaeoverse” (v. 1.4, 5), “rnaturalearth” (v. 1.0, 6), “rgbif” (v. 3.8, 7), “CoordinateCleaner” (v. 3.0, 8), “circlize” (v. 0.4, 9) and “gtools” (v. 3.9, 10) packages.

*Fossil Occurrence Data:*

To estimate the fossil distribution of each clade, we combined the observations recorded in the searched literature with occurrences from the Paleobiology Database (PBDB, 11), the world’s largest online repository for paleontological occurrence records. We synonymized the age ranges of the occurrences to the classification provided by the Geologic Time Scale 2020 (12). We removed any fossil occurrences with temporal uncertainty > 20 Ma and a spatial uncertainty > 0.1 degree, allowing us to estimate the paleolatitude of even the most temporally-uncertain records to within a radius of roughly 400-600 km (about five degrees of latitude), given the average speed of continental tectonic plates of about 2-3 cm per year (13). We only used fossil records associated with published literature or those that had a written history of identification, aging, location matching, and provenance. Given the structure of our literature search and the supplementary PBDB downloads, the fossil observations used were only those described in published, publicly available sources. These filters led to conservative estimates of where the taxon lived in the past. We then corrected the taxonomy of the observations; for clades above the family level, we ensured that every genus provided by the PDBD was properly identified as a member of the clade. For clades at the family level or below, we repeated this vetting at the level of individual species. Next, we searched the literature for phylogenies, removing taxa that were dubiously assigned the clade of interest. See Supplementary Table S1 for a comprehensive list of taxonomic adjustments in the fossil record. In general, we tended to be conservative when determining which taxa were considered part of the clade of interest; if the most recent published literature cast doubt upon the position of one taxon within the clade, we removed it.

The current locations of fossil occurrences are different from where the fossils were deposited, due to plate tectonics. We therefore reconstructed the coordinates of the fossil locality at the time it was deposited (14), using the palaeoverse R package (v. 1.4, 5) and five separate models of tectonic plate evolution (15–19). We chose these models and the rotation algorithms provided by palaeoverse because they use paleomagnetic reference frames, a necessity for estimating true paleolatitudes (14, 20, 21). The average of the minimum and maximum estimated ages for each fossil occurrence was used as the age of rotation. Different paleo-coordinate models can lead to substantially different coordinate reconstructions (14), so we conducted our analyses for each model separately (see SI Appendix II) and averaged them together to create a “consensus” set of paleo-coordinates.

*Modern Occurrence Data:*

We downloaded modern occurrence data from the Global Biodiversity Information Facility (GBIF), the world’s largest digital repository of occurrence points, with over 49.5 million records across the clades examined (22). Like all repositories of biodiversity data, GBIF has substantial spatial and temporal biases (23). GBIF records are often incomplete for lesser-studied taxa such as invertebrates (24). In addition, GBIF records are biased towards populated and urban areas in the Northern Hemisphere and biodiversity institutions, and the repository often includes dubious occurrences, occurrences linked to incorrect locations, and occurrences from captivity. Therefore, we conducted a comprehensive cleaning of each set of GBIF occurrences. First, we cleaned the data by removing occurrences with geospatial issues relating to the coordinates: invalid and out of range coordinates, coordinates with failed or suspected reprojections, those with invalid datums, presumed negated latitudes and longitudes, presumed swapped coordinates, and occurrences without a taxon match. We then removed occurrences marked as fossil specimens and those with no known date of observation or an observation date earlier than 1500 CE, ensuring that the occurrences were all of living or recently dead organisms. Observations that occurred at the same coordinates and within the same year were additionally removed, for easier computation. We also removed observations with uncertainty > 50 km.

Next, we corrected the taxonomy of the data, removing incorrect taxonomic identifications. We checked taxa with “doubtful” taxonomic status and, if necessary, checked each genus to ensure that its placement within the clade reflected the most recent systematics and phylogenetics. After the taxonomy was corrected for each dataset, we used the CoordinateCleaner package (v. 3.0, 8) to further vet the occurrences, removing occurrences associated with biodiversity institutions, country centroids, and country capitals, along with records more than 25 km outside of the boundaries of the country listed. Finally, we manually vetted the geographic locations of the occurrence data. We first checked the locality information for character strings relating to planted, domestic, or captive observations: “arboretum”, “dendrarium”, “aquafarm”, “hatchery”, “zoo”, “aquarium”, “aviary”, “herbarium”, “greenhouse”, “nursery”, “museum”, “captivity”, “domestic”, “feral”, “cultivated”, “planted”, “ornamental”, “farm”, and “botanic garden”, in English, Spanish, and French (the most common languages for localities in the database). We then visually scanned for geographic outliers, removing them if they were in countries well outside the known native range of the clades. We used expert range polygons from the International Union on Conservation of Nature (25) when available to ensure that the GBIF data were congruent and reasonable. For the species that did not have valid IUCN range polygons, we used a combination of other sources, including Plants of the World Online (26) and the literature we collected for each clade (Supplementary Appendix I).

To ensure that our modern data were representative of the modern ranges of the clade, every genus within the clade was required to have at least one occurrence point. For genera that had no occurrences from GBIF, we searched the literature for published locations, and if they had affiliated coordinates to within 0.1 degree, we added them to our neontological dataset. Two clades, the mammal family Equidae (horses) and the angiosperm genus *Quercus* (oak), were removed from the quantitative analyses of geographic discrepancies, because they have been domesticated/cultivated by humans for thousands of years. Therefore, we were unable to determine which occurrences in the fossil and modern records were native vs. human-influenced.

**Supplementary Appendix I: Literature Search and Number of Occurrences**

Supplementary Table S1: Table of every clade encountered during the literature review, with the reference used to identify the clade and the number of fossil and modern occurrences added. Superscripts next to the taxon name identify taxa in which taxonomic standardization was necessary.

| **Taxon** | **Fossil Occurrences** | **Total Modern Occurrences** | **Fossil References** | **Modern References** |
| --- | --- | --- | --- | --- |
| Acipenseridae | 130 | 3718 | (27) |  |
| Acroporidae | 2579 | 20290 | (28–35) |  |
| Ailuridae | 47 | 123 | (36–40) |  |
| Alangiaceae | 17 | 6425 | (41) |  |
| Aneuretinae | 20 | 8 | (42) |  |
| Aniliidae^1^ | 5 | 232 | (43) |  |
| Anomaluridae^2^ | 16 | 495 | (44–49) | (45) |
| Anseranatidae | 4 | 79639 | (50–52) |  |
| Aplodontiidae | 324 | 699 | (53–59) |  |
| Araucariaceae | 2004 | 9201 | (60, 61) |  |
| Archaeidae^3^ | 73 | 343 | (62–66) |  |
| Augilini^4^ | 1 | 5 | (67) | (68) |
| *Avicennia* | 16 | 56842 | (69–76) |  |
| Boganiidae^5^ | 2 | 16 | (77, 78) | (79, 80) |
| Brachionichthyinae^6^ | 3 | 311 | (81) |  |
| *Brasenia* | 74 | 7016 | (82) |  |
| Bucorvidae | 2 | 19627 | (50, 83, 84) |  |
| Camelidae^7^ | 2076 | 6918 | (85, 86) |  |
| *Campanile* | 123 | 175 | (87–89) |  |
| Ceratodontiformes^8^ | 221 | 1236 | (90–92) |  |
| Chaeteessidae^9^ | 10 | 17 | (93–95) |  |
| Coleorrhyncha | 127 | 258 | (96) | (97) |
| Coliiformes^10^ | 18 | 129179 | (98–101) |  |
| Coracii | 31 | 370991 | (102–104) |  |
| Corytophanidae^11^ | 37 | 18467 | (105, 106) |  |
| Crocodilia | 3030 | 108381 | (107–114) |  |
| Ctenodactylidae | 266 | 327 | (115–121) |  |
| Cycadales | 2243 | 21613 | (122–132) |  |
| Cyclopedidae | 4 | 358 | (133) |  |
| Dasyproctidae | 36 | 14893 | (134) |  |
| *Diagrypnodes* | 1 | 9 | (135) |  |
| Diatomyidae | 41 | 39 | (136–141) |  |
| Elephantidae | 1549 | 18748 | (142–144) |  |
| Eomeropidae | 17 | 4 | (145–151) |  |
| Equidae | X | X | (152, 153) |  |
| Erethizontidae | 138 | 19335 | (154–157) |  |
| *Euryale* | 13 | 229 | (82) |  |
| *Gackstroemia* | 1 | 803 | (158) |  |
| Ginkgoaceae | 851 | 467 | (159) |  |
| Giraffidae | 690 | 12469 | (160–162) |  |
| Glossinidae | 8 | 317 | (163) |  |
| Gruidae | 92 | 1900349 | (102, 164) |  |
| Hamamelidaceae^12^ | 137 | 30178 | (165–169) |  |
| *Hippopotamus* | 491 | 6954 | (170, 171) |  |
| Huttoniidae | 4 | 7 | (172) |  |
| Hyaenidae | 732 | 12009 | (173, 174) |  |
| Hyriidae | 36 | 4063 | (175) |  |
| Ironomyiidae | 40 | 44 | (176, 177) |  |
| *Itea* | 51 | 7755 | (178–200) |  |
| Lepisosteidae^13^ | 648 | 54340 | (107, 201–210) |  |
| Leptomyrmex | 6 | 3448 | (93, 211, 212) |  |
| Leptosomiformes^14^ | 10 | 1524 | (213–215) |  |
| Lophopidae^15^ | 6 | 818 | (216) | (217) |
| Macroscelidea | 864 | 1025 | (218) |  |
| Malpighiaceae | 36 | 90817 | (219–222) |  |
| Mantophasmatidae^16^ | 17 | 59 | (223) | (224–227) |
| Mastotermitidae | 35 | 210 | (42, 228–230) |  |
| Megadermatidae | 31 | 1776 | (231) |  |
| Megalyridae^17^ | 69 | 167 | (232) | (233) |
| Microbiotheria | 75 | 572 | (234) |  |
| Musophagiformes^18^ | 3 | 118752 | (213) |  |
| Myrmeciinae | 42 | 19302 | (42, 235–239) |  |
| Myzopodidae | 3 | 36 | (240) |  |
| Nannochoristidae | 30 | 196 | (241, 242) |  |
| Nesomyidae | 70 | 2817 | (243–246) |  |
| Notonemouridae^19^ | 3 | 2566 | (247) | (248) |
| Nyctibiidae^20^ | 4 | 39571 | (50, 249–251) |  |
| Nymphidae | 37 | 2073 | (252, 253) |  |
| *Nypa*^21^ | 210 | 1243 | (69, 70, 73, 76, 254–264) |  |
| Nyssaceae | 200 | 13286 | (72, 74, 75, 265) |  |
| Ochotonidae | 773 | 9916 | (266–268) |  |
| Ogcocephalidae | 8 | 8323 | (81) |  |
| Ommatidae | 170 | 16 | (269–273) |  |
| Opilioacarida^22^ | 2 | 150 | (274) | (275, 276) |
| Opisthocomiformes | 4 | 15791 | (277) |  |
| Ornithorhynchidae | 11 | 15631 | (278, 279) |  |
| *Panthera* | 425 | 19309 | (280–282) |  |
| Pedetidae | 28 | 299 | (283) |  |
| Perissommatidae | 12 | 6 | (284) |  |
| *Phimophorus*^23^ | 1 | 1 | (285) | (286) |
| *Pholadomya* | 1678 | 4 | (287–291) |  |
| Pholidota | 24 | 853 | (292–294) |  |
| Phreatoicidea^24^ | 8 | 1226 | (295, 296) | (297, 298) |
| Phylliidae | 1 | 383 | (299) |  |
| Phytocreneae^25^ | 34 | 2428 | (300, 301) | (301) |
| Podargiformes | 3 | 97587 | (50, 302) |  |
| Polychrotidae^26^ | 4 | 1462 | (106, 303) | (303) |
| *Porites* | 2001 | 18523 | (34, 304–306) |  |
| Psittaciformes | 282 | 1660548 | (50, 307–312) |  |
| *Pterocarya* | 148 | 1755 | (72, 74, 75, 313, 314) |  |
| *Quercus* | X | X | (315–319) |  |
| Rhinocerotidae | 2566 | 4646 | (320, 321) |  |
| Rhizophoraceae | 81 | 89545 | (69, 70, 73, 76) |  |
| *Rhopica* | 1 | 19 | (93) | (322) |
| Rhynchocephalia | 134 | 1109 | (323–331) |  |
| Roridulaceae | 1 | 263 | (332) |  |
| Sabiaceae | 89 | 11561 | (333, 334) |  |
| Sagittariidae | 2 | 25803 | (50, 335) |  |
| *Saiga* | 57 | 104 | (336–348) |  |
| *Sellnickiella* | 1 | 2 | (349) |  |
| Siganidae | 12 | 18850 | (350–352) |  |
| Solifugae | 16 | 5356 | (274) |  |
| Steatornithidae^27^ | 2 | 2354 | (353) |  |
| Struthionidae | 117 | 44283 | (50, 354–356) |  |
| Synlestidae | 8 | 5117 | (357) |  |
| Synxenidae^28^ | 6 | 257 | (358) | (359) |
| Tapiridae | 513 | 7442 | (360) |  |
| Tarsiidae | 8 | 507 | (361) |  |
| *Tetraclinis*^29^ | 6 | 604 | (362) | (362) |
| Tettigarctidae | 44 | 70047 | (363–366) |  |
| Theaceae^30^ | 179 | 17767 | (367, 368) | (369) |
| Thryonomyidae | 103 | 652 | (370–373) |  |
| Timematidae | 6 | 315 | (42, 374) |  |
| Todidae | 5 | 18359 | (375) |  |
| Tragulidae | 263 | 805 | (376–378) |  |
| Trigoniidae | 1616 | 1013 | (379) |  |
| Trochilidae | 11 | 2736544 | (50, 213, 380, 381) |  |
| Trogonidae | 8 | 256158 | (382–386) |  |
| Tubulidentata | 104 | 674 | (387–390) |  |
| Ursidae | 1013 | 57324 | (391–393) |  |
| Varanidae^31^ | 252 | 70047 | (394) |  |
| *Vauquelinia* | 12 | 1150 | (395) |  |
| Xyelidae | 140 | 106 | (396, 397) |  |

1. Aniliidae: removed all fossil genera except *Borealoilysia* and *Australophis*, per (43)
2. Anomaluridae: removed fossil *Zenkerella, Nonanomalurus, Nementchamys, Oromys, Prozenkerella, Pondaungimys*; modern *Zenkerella* from family, per (45)
3. Archaeidae: removed fossil *Lacunauchenius*, per (62). Removed modern genus *Archea* (unclear taxonomy).
4. Augilini: added *Youtuus* type specimens to modern occurrences (68)
5. Boganiidae: Removed modern *Hobartius* and *Hydnobioides*, per (80), added *Afroboganium* type specimens to modern occurrences (79).
6. Brachionichthyinae: Used Brachionichthyidae for fossil occurrences (398).
7. Camelidae: Removed *Blancocamelus, Camelops, Hemiauchenia* from modern occurrences (extinct taxa).
8. Ceratodontiformes: Removed *Ceratodus* from modern occurrences.
9. Chaeteessidae: Removed *Chaetessa burmeisteri:* reassigned to Mantoida (94)
10. Coliiformes: Removed *Eocolius walkeri*, per (100)
11. Corytophanidae: *Geiseltaliellus* added to clade (105)
12. Hamamelidaceae: removed *Liquidambar, Protoaltingia* from occurrences, per (165)
13. Lepisosteidae: added *Nhanulepisosteus* from fossil occurrences (202)
14. Leptosomiformes: added *Plesiocathartes* to fossil occurrences (214)
15. Lophopidae: removed *Oenopiella* from modern occurrences, per (217)
16. Mantophasmatidae: added *Viridiphasma*, *Pachyphasma, Striatophasma,* Kuboesphasma, and Minutophasma type specimens to modern occurrences (224–226).
17. Megalyridae: added *Cryptalyra* type specimen to modern occurrences (233).
18. Musophagiformes: added *Foro* to fossil occurrences, per (213).
19. Notonemouridae: added *Otehiwi* type specimen to modern occurrences (248).
20. Nyctibiidae: added *Paraprefica* to fossil occurrences, per (249, 250).
21. *Nypa*: Added *Spinizonicolpites* (fossil pollen) to fossil occurrences, per (260)
22. Opilioacarida: added type specimens of *Brasilacarus* and *Indiacarus* (275, 276).
23. Phimophorus: added record of *Phimophorus spissicornis* (286).
24. Phreatoicidea: added type specimens of *Andhracoides* (297, 298).
25. Phytocreneae: Trimmed fossil occurrences of Icacinaceae to genera within subfamily using (301): *Pyrenacantha, Phytocrene, Paleophytocrene, Natsiatum* had fossil occurrences. Used (301) to limit modern occurrence searches.
26. Polychrotidae: Removed *Anolis* from clade and *Phenacosaurus* from the modern records, per (303).
27. Steatornithidae: *Euronyctibius* added to clade.
28. Synxenidae: Added type specimens for *Condexenus* to modern occurrences (359).
29. Tetraclinis: Merged with some *Libocedrus* species, per (362).
30. Theaceae: Removed “*Fresiera*” (unclear genus), *Ternstroemia* (369)
31. Varanidae: Added *Saniwa*, *Ovoo, Aiolosaurus, Paravaranus, Proplatynotia*, and removed *Cherminotus* from fossil occurrences, per (394)

**Other References Used in the Literature Search: 395-478**

**Supplementary Appendix II: Influence of Paleo-Coordinate Models**

Several distinct models have been developed that account for continental drift when estimating where a fossil location was deposited. These different paleo-coordinate models lead to different estimates of where on Earth’s surface taxa lived in the past (14). We averaged five models of tectonic plate evolution (15–19) together to estimate the paleo-latitude and paleo-longitude of the fossil occurrences. This method can mitigate outlier coordinates provided by any one model, but to ensure that our results are not influenced by our choice of paleo-coordinate models, we compared our results when each of the five different models were used. We removed all clades with total discrepancy < 1000 km or latitudinal discrepancies < 500 km, leaving the 120 clades we described in our main analysis. One additional clade (*Gackstroemia*) had no resolvable paleo-coordinates using the PaleoMap model (18), and the lone observation of *Phimophorus* did not allow us to construct a convex hull for the discrepancy analyses, so those two clades were removed from this comparison. The discrepancies between fossil and modern occurrence for each of the remaining 118 clades were highly correlated across the five models and the two methods of discrepancy calculation (total discrepancy, Spearman’s ρ > 0.984; latitudinal discrepancy, ρ > 0.951; Figure S1). However, in some clades the calculated latitudinal discrepancy values were substantially different across the different models. For example, the total discrepancy values of Timematidae ranged from 8751.6 km (18) to 12492 km (15). The standard deviation of the total discrepancy between fossil and modern distributions across the five models ranged from 0 km (Elephantidae) to 1399 km (Timematidae), with an average of 311.4 km. For latitudinal discrepancy, standard deviations ranged from 0 km (in the case of no latitudinal change) to 1464 km (Hyriidae). As expected given the uncertain nature of continental drift, older clades had substantially higher standard deviations than newer clades (total discrepancy, Spearman’s ρ = 0.513; latitudinal discrepancy, Spearman’s ρ = 0.494).

The differences in latitudinal discrepancy across the five models were minute enough that they did not substantially affect the mean and standard deviations for either total discrepancy (mean: 7943 – 8043 km; standard deviation: 4681 – 4819 km) or latitudinal discrepancy (mean: 4045 – 4127 km; standard deviation: 2985 –3227 km). Similarly, the taxonomic rank of the clades did not significantly affect the magnitude of the total (Kruskal-Wallis χ^2^ < 8.29, df = 4, p > 0.08) and latitudinal discrepancy (KW χ^2^ < 1.11, df = 4, p > 0.89) for any paleo-coordinate models. The insubstantial changes and strong correlations between the models in these data suggest that our overall results and the interpretation of them would be similar, regardless of which model we used to calculate the paleolatitude of the fossils. A dataset comparing the discrepancy values of the different models is provided in the online data repository (PRIVATE LINK: https://figshare.com/s/0c706eca7ba1d21deba0).

Figure S1: Pairwise correlation matrix of the total discrepancy (a) and latitudinal discrepancy (b) values calculated from the five paleo-coordinate models used in this analysis. Spearman’s rank correlation coefficients are listed for each correlation.

**Supplementary Appendix III: False Absences and the Fossil Record**

The fossil record is inherently incomplete and discontinuous in time. Most species that ever lived were never fossilized, and for those that were, their fossil records do not represent the entire geographic or environmental range of the taxon. As a result, false absences likely abound in the fossil record. This potential for false absences in the fossil record means that dynamic processes like geographic change cannot be fully described. For example, Figure S2 shows two hypothesized geographic ranges in the past based on the fossil record (grey dots). H1 (top left) assumes that there are no false absences, and therefore suggests that the geographic discrepancy between the fossil and modern record to due to a complete shift from one region to another. In contrast, H2 (bottom left) incorporates false absences (grey x’s), suggesting that the discrepancy was caused by a range contraction rather than a complete geographical shift. These two hypotheses describe different processes, but end with the same result (right). Because we can never be sure that the fossil record we have is complete, we cannot determine which of these two mechanisms caused this discrepancy.

Figure S2: Conceptual figure describing the influence of the incomplete fossil record on the interpretation of range dynamics. Left: two hypotheses describing the past range of a species: H1 assumes that the fossil record (grey dots) is well-sampled, whereas H2 assumes there are many false absences (grey x’s). Therefore, the discrepancy between the modern range (blue polygon) and the fossil record is explained differently by the two hypotheses. Because the fossil record is incomplete, we cannot falsify either of these hypotheses.

**Legend for Table S2:**

A table of all clades discovered in the literature review (n = 123), with the name of the clade and its higher taxonomic identity (“Class”, “Higher Taxon”). Taxon Level = the taxonomic rank of the clade (e.g., family, suborder). Oldest = the GTS2020 calibrated age of the oldest fossil specimen. Newest = the GTS2020 calibrated age of the newest fossil specimen. Pleistocene = whether the geographic discrepancy observed is more recent than the beginning of the Pleistocene (< 2.58 mya), where “P” means that the largest geographic discrepancy occurred before the Pleistocene. Total Discrepancy = the calculated total discrepancy for each clade (see main text). Latitudinal Discrepancy = the calculated latitudinal discrepancy for each clade (see main text).

**Supplementary Reference List**

(36, 37, 41–46, 53–56, 59, 62, 66–68, 78–80, 83, 84, 87, 90–92, 94, 97–100, 102–105, 107–109, 114, 121–127, 130, 132, 135–138, 147, 148, 154, 155, 158, 160, 161, 166, 167, 177, 178, 181, 185, 187, 192, 193, 195, 197–199, 201–204, 208–213, 218–221, 223, 224, 228, 233–238, 240, 241, 243–247, 250, 252, 253, 269, 271, 272, 274, 275, 285–289, 293–295, 308, 312, 315–318, 322, 325–329, 331, 337–346, 354, 356, 357, 360, 362, 371–374, 376, 380, 386, 387, 390, 395, 396, 399–433) **and** (27, 38–40, 47–52, 57, 58, 60, 61, 63–65, 77, 82, 85, 86, 88, 89, 93, 95, 96, 101, 106, 110–113, 115–119, 128, 129, 131, 133, 139–141, 145, 146, 149–151, 156, 157, 159, 162–165, 168, 169, 172, 175, 176, 179, 180, 182–184, 186, 188–191, 194, 196, 200, 205–207, 214–216, 222, 225, 226, 229, 230, 232, 239, 242, 248, 249, 251, 265, 270, 273, 276, 277, 284, 290–292, 296–303, 307, 309–311, 313, 314, 319, 323, 324, 330, 332–336, 347–349, 353, 355, 358, 359, 361, 367, 368, 370, 375, 377–379, 381–385, 388, 389, 391–393, 397, 434–478) **and** (227)

1.	N. Cohen, T. Arieli, Field research in conflict environments: Methodological challenges and snowball sampling. *J. Peace Res.* **48**, 423–435 (2011).

2.	R Core Team, R: A Language and Environment for Statistical	Computing. (2025). Deposited 2025.

3.	R. J. Hijmans, terra: Spatial Data Analysis. (2022). Deposited 2022.

4.	J. T. Flannery-Sutherland, N. B. Raja, Á. T. Kocsis, W. Kiessling, fossilbrush: An R package for automated detection and resolution of anomalies in palaeontological occurrence data. *Methods Ecol. Evol.* **13**, 2404–2418 (2022).

5.	L. A. Jones, *et al.*, palaeoverse: A community-driven R package to support palaeobiological analysis. *Methods Ecol. Evol.* **14**, 2205–2215 (2023).

6.	P. Massicotte, A. South, rnaturalearth: World Map Data from	Natural Earth. (2023). Deposited 2023.

7.	S. Chamberlain, *et al.*, rgbif: Interface to the Global Biodiversity Information	Facility API. (2025). Deposited 2025.

8.	A. Zizka, *et al.*, CoordinateCleaner: Standardized cleaning of occurrence records from biological collection databases. *Methods Ecol. Evol.* **10**, 744–751 (2019).

9.	Z. Gu, L. Gu, R. Eils, M. Schlesner, B. Brors, circlize Implements and enhances circular visualization in R. *Bioinforma. Oxf. Engl.* **30**, 2811–2812 (2014).

10.	G. R. Warnes, *et al.*, gtools: Various R Programming Tools. (2023). Deposited 20 November 2023.

11.	The Paleobiology Database. Available at: http://paleobiodb.org/data1.2/occs/list.txt?datainfo&rowcount&max_ma=400&min_ma=0 [Accessed 19 February 2025].

12.	F. M. Gradstein, J. G. Ogg, M. D. Schmitz, G. M. Ogg, Eds., *Geologic Time Scale 2020* (Elsevier BV, 2020).

13.	S. Zahirovic, R. D. Müller, M. Seton, N. Flament, Tectonic speed limits from plate kinematic reconstructions. *Earth Planet. Sci. Lett.* **418**, 40–52 (2015).

14.	M. Seton, S. E. Williams, M. Domeier, A. S. Collins, K. Sigloch, Deconstructing plate tectonic reconstructions. *Nat. Rev. Earth Environ.* **4**, 185–204 (2023).

15.	N. Wright, S. Zahirovic, R. D. Müller, M. Seton, Towards community-driven paleogeographic reconstructions: integrating open-access paleogeographic and paleobiology data with plate tectonics. *Biogeosciences* **10**, 1529–1541 (2013).

16.	K. J. Matthews, *et al.*, Global plate boundary evolution and kinematics since the late Paleozoic. *Glob. Planet. Change* **146**, 226–250 (2016).

17.	T. H. Torsvik, L. R. M. Cocks, *Earth History and Palaeogeography* (Cambridge University Press, 2016).

18.	C. R. Scotese, N. M. Wright, PALEOMAP Paleodigital Elevation Models (PaleoDEMs) for the Phanerozoic. Deposited 2018.

19.	A. S. Merdith, *et al.*, Extending full-plate tectonic models into deep time: Linking the Neoproterozoic and the Phanerozoic. *Earth-Sci. Rev.* **214**, 103477 (2021).

20.	T. H. Torsvik, R. D. Müller, R. Van der Voo, B. Steinberger, C. Gaina, Global plate motion frames: Toward a unified model. *Rev. Geophys.* **46** (2008).

21.	L. Buffan, *et al.*, Mind the uncertainty: Global plate model choice impacts deep-time palaeobiological studies. *Methods Ecol. Evol.* **14**, 3007–3019 (2023).

22.	A. Telenius, Biodiversity information goes public: GBIF at your service. *Nord. J. Bot.* **29**, 378–381 (2011).

23.	E. H. Boakes, *et al.*, Distorted views of biodiversity: spatial and temporal bias in species occurrence data. *PLOS Biol.* **8**, e1000385 (2010).

24.	J. Beck, L. Ballesteros‐Mejia, P. Nagel, I. J. Kitching, Online solutions and the ‘Wallacean shortfall’: what does GBIF contribute to our knowledge of species’ ranges? *Divers. Distrib.* **19**, 1043–1050 (2013).

25.	IUCN, *The IUCN Red List of Threatened Species.*, 2019th–3. Ed. (2020).

26.	POWO, *Plants of the World Online* (Royal Botanic Gardens, Kew, 2025).

27.	D. M. Martill, A sturgeon (Actinopterygii, Acipenseriformes) from the Upper Cretaceous of Africa. *Cretac. Res.* **148**, 105546 (2023).

28.	C. C. Wallace, F. R. Bosellini, Acropora (Scleractinia) from the Oligocene and Miocene of Europe: species longevity, origination and turnover following the Eocene–Oligocene transition. *J. Syst. Palaeontol.* **13**, 447–469 (2015).

29.	C. C. Wallace, B. R. Rosen, Diverse staghorn corals (Acropora) in high-latitude Eocene assemblages: implications for the evolution of modern diversity patterns of reef corals. *Proc. R. Soc. B Biol. Sci.* **273**, 975–982 (2006).

30.	C. C. Wallace, New species and records from the Eocene of England and France support early diversification of the coral genus Acropora. *J. Paleontol.* **82**, 313–328 (2008).

31.	C. C. Wallace, R. W. Portell, Earliest western Atlantic staghorn corals (Acropora) from the lower Oligocene Suwannee Limestone of Florida, USA, and their significance for modern coral distribution. *J. Paleontol.* **96**, 1390–1399 (2022).

32.	N. Santodomingo, C. C. Wallace, K. G. Johnson, Fossils reveal a high diversity of the staghorn coral genera Acropora and Isopora (Scleractinia: Acroporidae) in the Neogene of Indonesia. *Zool. J. Linn. Soc.* **175**, 677–763 (2015).

33.	A. F. Budd, C. C. Wallace, First Record of the Indo-Pacific Reef Coral Genus Isopora in the Caribbean Region: Two New Species from the Neogene of Curaçao, Netherlands Antilles. *Palaeontology* **51**, 1387–1401 (2008).

34.	T. C. Brachert, *et al.*, Slow-growing reef corals as climate archives: A case study of the Middle Eocene Climatic Optimum 40 Ma ago. *Sci. Adv.* **8**, eabm3875 (2022).

35.	W. Renema, *et al.*, Are coral reefs victims of their own past success? *Sci. Adv.* **2**, e1500850 (2016).

36.	J. Baskin, E. Dickinson, J. DuBois, H. Galiano, A. Hartstone-Rose, ?Amphictis (Carnivora, Ailuridae) from the Belgrade Formation of North Carolina, USA. *PeerJ* **8**, e9284 (2020).

37.	G. D. Koufos, “The Fossil Record of Ailurids (Mammalia: Carnivora: Ailuridae) in Greece” in *Fossil Vertebrates of Greece Vol. 2: Laurasiatherians, Artiodactyles, Perissodactyles, Carnivorans, and Island Endemics*, E. Vlachos, Ed. (Springer International Publishing, 2022), pp. 629–633.

38.	S. Ogino, *et al.*, Mandible and Lower Dentition of Parailurus baikalicus (Ailuridae, Carnivora) from Transbaikal area, Russia. *Paleontol. Res.* **13**, 259–264 (2009).

39.	N. Spassov, D. Geraads, A skull of Simocyon primigenius (Roth & Wagner, 1854) (Carnivora, Ailuridae) from the late Miocene of Karaslari (Republic of Macedonia), with remarks on the systematics and evolution of the genus. *Neues Jahrb. Für Geol. Paläontol. - Abh.* 151–161 (2011). https://doi.org/10.1127/0077-7749/2011/0188.

40.	S. C. Wallace, L. M. Lyon, “Chapter 3 - Systematic revision of the Ailurinae (Mammalia: Carnivora: Ailuridae): with a new species from North America” in *Red Panda (Second Edition)*, A. R. Glatston, Ed. (Academic Press, 2022), pp. 31–52.

41.	C.-M. Feng, S. R. Manchester, Q.-Y. (Jenny) Xiang, Phylogeny and biogeography of Alangiaceae (Cornales) inferred from DNA sequences, morphology, and fossils. *Mol. Phylogenet. Evol.* **51**, 201–214 (2009).

42.	P. Barden, J. L. Ware, Relevant Relicts: The Impact of Fossil Distributions on Biogeographic Reconstruction. *Insect Syst. Divers.* **1**, 73–80 (2017).

43.	J. J. Head, A South American snake lineage from the Eocene Greenhouse of North America and a reappraisal of the fossil record of “anilioid” snakes. *Geobios* **66–67**, 55–65 (2021).

44.	P. M. C. Coster, K. C. Beard, M. J. Salem, Y. Chaimanee, J.-J. Jaeger, New fossils from the Paleogene of central Libya illuminate the evolutionary history of endemic African anomaluroid rodents. *Front. Earth Sci.* **3** (2015).

45.	P.-H. Fabre, *et al.*, Flightless scaly-tailed squirrels never learned how to fly: A reappraisal of Anomaluridae phylogeny. *Zool. Scr.* **47**, 404–417 (2018).

46.	S. Heritage, *et al.*, Ancient phylogenetic divergence of the enigmatic African rodent Zenkerella and the origin of anomalurid gliding. *PeerJ* **4**, e2320 (2016).

47.	L. Marivaux, Adnet ,Sylvain, Benammi ,Mohamed, Tabuce ,Rodolphe, M. and Benammi, Anomaluroid rodents from the earliest Oligocene of Dakhla, Morocco, reveal the long-lived and morphologically conservative pattern of the Anomaluridae and Nonanomaluridae during the Tertiary in Africa. *J. Syst. Palaeontol.* **15**, 539–569 (2017).

48.	H. M. Sallam, E. R. Seiffert, E. L. Simons, A highly derived anomalurid rodent (Mammalia) from the earliest Late Eocene of Egypt. *Palaeontology* **53**, 803–813 (2010).

49.	E. R. Seiffert, *et al.*, Oldest record of a crown anomaluroid rodent from sub-Saharan Africa: a new genus and species from the early Oligocene Topernawi Formation of northern Kenya. *Hist. Biol.* 1–11 (2024). https://doi.org/10.1080/08912963.2024.2370015.

50.	G. Mayr, Two-phase extinction of “Southern Hemispheric” birds in the Cenozoic of Europe and the origin of the Neotropic avifauna. *Palaeobiodiversity Palaeoenvironments* **91**, 325–333 (2011).

51.	T. H. Worthy, J. D. Scanlon, An Oligo-Miocene magpie goose (Aves: Anseranatidae) from Riversleigh, northwestern Queensland, Australia. *J. Vertebr. Paleontol.* **29**, 205–211 (2009).

52.	C. Mourer-Chauviré, D. Berthet, M. Hugueney, The late Oligocene birds of the Créchy quarry (Allier, France), with a description of two new genera (Aves: Pelecaniformes: Phalacrocoracidae, and Anseriformes: Anseranatidae). *Senckenberg. Lethaea* **84**, 303–315 (2004).

53.	G. Daxner-Höck, T. Mörs, I. Filinov, A. Shchetnikov, M. A. Erbajeva, Geology and lithology of the Tagay-1 section at Olkhon Island (Lake Baikal, Eastern Siberia), and description of Aplodontidae, Mylagaulidae and Sciuridae (Rodentia, Mammalia). *Palaeobiodiversity Palaeoenvironments* **102**, 843–857 (2022).

54.	S. S. B. Hopkins, Causes of lineage decline in the Aplodontidae: Testing for the influence of physical and biological change. *Palaeogeogr. Palaeoclimatol. Palaeoecol.* **246**, 331–353 (2007).

55.	S. S. B. Hopkins, Phylogeny and biogeography of the genus Ansomys Qiu, 1987 (Mammalia: Rodentia: Aplodontidae) and description of a new species from the barstovian (mid-Miocene) of Montana. *J. Paleontol.* **78**, 731–740 (2004).

56.	S. S. B. Hopkins, Phylogeny, systematics, and evolution of hypsodonty in the Aplodontiinae (Mammalia, Rodentia, Aplodontiidae), with the description of several new species. *J. Vertebr. Paleontol.* **39**, e1668401 (2019).

57.	O. Maridet, G. Daxner-Höck, P. López-Guerrero, U. B. Göhlich, The record of Aplodontidae (Rodentia, Mammalia) in the Oligocene and Miocene of the Valley of Lakes (Central Mongolia) with some comments on the morphologic variability. *Palaeobiodiversity Palaeoenvironments* **97**, 25–49 (2017).

58.	M. Vianey-Liaud, H. Gomes Rodrigues, L. Marivaux, Early adaptive radiations of Aplodontoidea (Rodentia, Mammalia) on the Holarctic region: systematics, and phylogenetic and paleobiogeographic implications. *Paläontol. Z.* **87**, 83–120 (2013).

59.	S. S. B. Hopkins, Phylogeny and evolutionary history of the Aplodontoidea (Mammalia: Rodentia). *Zool. J. Linn. Soc.* **153**, 769–838 (2008).

60.	Z. Shaolin, Z. Lidong, Z. Wu, Y. Yajun, A new female cone, Araucaria beipoiaoensis sp. nov. from the Middle Jurassic Tiaojishan Formation, Beipiao, Western Liaoning, China and Its evolutionary significance. *Acta Geol. Sin. - Engl. Ed.* **82**, 266–282 (2008).

61.	R. A. Stockey, G. W. Rothwell, Diversification of crown group Araucaria: the role of Araucaria famii sp. nov. in the Late Cretaceous (Campanian) radiation of Araucariaceae in the Northern Hemisphere. *Am. J. Bot.* **107**, 1072–1093 (2020).

62.	B. Carbuccia, H. M. Wood, C. Rollard, A. Nel, R. Garrouste, A new Myrmecarchaea (Araneae: Archaeidae) species from Oise amber (earliest Eocene, France). *Bull. Société Géologique Fr.* **191**, 24 (2020).

63.	H. M. Wood, N. J. Matzke, R. G. Gillespie, C. E. Griswold, Treating fossils as terminal taxa in divergence time estimation reveals ancient vicariance patterns in the palpimanoid spiders. *Syst. Biol.* **62**, 264–284 (2013).

64.	H. M. Wood, Integrating fossil and extant lineages: an examination of morphological space through time (Araneae: Archaeidae). *J. Arachnol.* **45**, 20–29 (2017).

65.	E. E. Saupe, P. A. Selden, First fossil Mecysmaucheniidae (Arachnida, Chelicerata, Araneae), from Lower Cretaceous (Uppermost Albian) amber of Charente-Maritime, France. *Geodiversitas* **31**, 49–60 (2009).

66.	K. Eskov, Archaeid spiders from Eocene Baltic amber (Chelicerata: Araneida: Archaeidae) with remarks on the so-called “Gondwanan” ranges of Recent taxa. *Neues Jahrb. Für Geol. Paläontol. - Abh.* 311–328 (1992). https://doi.org/10.1127/njgpa/185/1992/311.

67.	T. Bourgoin, R.-R. Wang, V. M. Gnezdilov, First fossil record of Caliscelidae (Hemiptera: Fulgoroidea): a new Early Miocene Dominican amber genus extends the distribution of Augilini to the Neotropics. *J. Syst. Palaeontol.* **14**, 211–218 (2016).

68.	N. Gong, L. Yang, X.-S. Chen, Youtuus, a new bamboo-feeding genus of the tribe Augilini with two new species from China (Hemiptera, Fulgoromorpha, Caliscelidae). *ZooKeys* **783**, 85–96 (2018).

69.	J.-C. Plaziat, C. Cavagnetto, J.-C. Koeniguer, F. Baltzer, History and biogeography of the mangrove ecosystem, based on a critical reassessment of the paleontological record. *Wetl. Ecol. Manag.* **9**, 161–180 (2001).

70.	J. Srivastava, V. Prasad, Evolution and paleobiogeography of mangroves. *Mar. Ecol.* **40**, e12571 (2019).

71.	G. Suan, *et al.*, Subtropical climate conditions and mangrove growth in Arctic Siberia during the early Eocene. *Geology* **45**, 539–542 (2017).

72.	D. Biltekin, *et al.*, Anatolia: A long-time plant refuge area documented by pollen records over the last 23   million years. *Rev. Palaeobot. Palynol.* **215**, 1–22 (2015).

73.	D. A. Friess, *et al.*, The State of the World’s Mangrove Forests: Past, Present, and Future. *Annu. Rev. Environ. Resour.* **44**, 89–115 (2019).

74.	G. Jiménez-Moreno, S. Fauquette, J.-P. Suc, Vegetation, climate and palaeoaltitude reconstructions of the Eastern Alps during the Miocene based on pollen records from Austria, Central Europe. *J. Biogeogr.* **35**, 1638–1649 (2008).

75.	G. Jiménez-Moreno, S. Fauquette, J.-P. Suc, Miocene to Pliocene vegetation reconstruction and climate estimates in the Iberian Peninsula from pollen data. *Rev. Palaeobot. Palynol.* **162**, 403–415 (2010).

76.	A. M. Ellison, E. J. Farnsworth, R. E. Merkt, Origins of mangrove ecosystems and the mangrove biodiversity anomaly. *Glob. Ecol. Biogeogr.* **8**, 95–115 (1999).

77.	Z. Liu, A. Ślipiński, J. F. Lawrence, D. Ren, H. Pang, Palaeoboganium gen. nov. from the Middle Jurassic of China (Coleoptera: Cucujoidea: Boganiidae): the first cycad pollinators? *J. Syst. Palaeontol.* **16**, 351–360 (2018).

78.	C. Cai, D. Huang, First boganiine beetle in mid-Cretaceous amber from northern Myanmar (Coleoptera: Boganiidae). *Proc. Geol. Assoc.* **130**, 81–86 (2019).

79.	P. Jałoszyński, A. Ślipiński, Afroboganium wakefieldense sp. n. from KwaZulu Natal, South Africa (Coleoptera: Boganiidae: Boganiinae). *Zootaxa* **4995**, 382–388 (2021).

80.	H. E. Escalona, J. F. Lawrence, M. Wanat, A. Ślipiński, Phylogeny and placement of Boganiidae (Coleoptera, Cucujoidea) with a review of Australian and New Caledonian taxa. *Syst. Entomol.* **40**, 628–651 (2015).

81.	G. Carnevale, T. W. Pietsch, Batfishes from the Eocene of Monte Bolca. *Geol. Mag.* **148**, 461–472 (2011).

82.	C. Löhne, *et al.*, Biogeography of Nymphaeales: extant patterns and historical events. *Taxon* **57**, 1123–19E (2008).

83.	Z. Boev, D. Kovachev, Euroceros bulgaricus gen. nov., sp. nov. from Hadzhidimovo (SW Bulgaria) (Late Miocene) – the first European record of Hornbills (Aves: Coraciiformes). *Geobios* **40**, 39–49 (2007).

84.	J. Brunet, Oiseaux miocenes de Beni-Mellal (Maroc); un complement it leur etude. *Notes Memoires Serv. Mines Carte Geol. Maroc* **237**, 109–111 (1971).

85.	A. H. Marín-Leyva, *et al.*, Environmental inferences based on the dietary ecology of camelids from west-central Mexico during the Late Pleistocene. *Hist. Biol.* **35**, 1011–1027 (2023).

86.	S. D. Webb, J. Meachen, On the origin of Lamine Camelidae including a new genus from the Late Miocene of the High Plains. *Bull. Carnegie Mus. Nat. Hist.* **2004**, 349–362 (2004).

87.	N. J. de Winter, *et al.*, The giant marine gastropod Campanile giganteum (Lamarck, 1804) as a high-resolution archive of seasonality in the Eocene Greenhouse World. *Geochem. Geophys. Geosystems* **21**, e2019GC008794 (2020).

88.	R. W. Portell, S. K. Donovan, Campanile trevorjacksoni sp. nov. (Mollusca: Gastropoda) from the Eocene of Jamaica: at last, a name for the first fossil used in intercontinental biostratigraphic correlation (de la Beche 1827). *Geol. J.* **43**, 542–551 (2008).

89.	S. F. Mitchell, Discussion of Campanile trevorjacksoni sp. nov. (Mollusca: Gastropoda) from the Eocene of Jamaica—at last, a name for the first fossil used in intercontinental biostratigraphic correlation (de la Beche 1827): (v. 43, p. 542–551). *Geol. J.* **44**, 494–496 (2009).

90.	C. D. Brownstein, R. C. Harrington, T. J. Near, The biogeography of extant lungfishes traces the breakup of Gondwana. *J. Biogeogr.* **50**, 1191–1198 (2023).

91.	A. Kemp, L. Cavin, G. Guinot, Evolutionary history of lungfishes with a new phylogeny of post-Devonian genera. *Palaeogeogr. Palaeoclimatol. Palaeoecol.* **471**, 209–219 (2017).

92.	J. A. Frederickson, R. L. Cifelli, New Cretaceous lungfishes (Dipnoi, Ceratodontidae) from western North America. *J. Paleontol.* **91**, 146–161 (2017).

93.	J. Rust, *et al.*, Biogeographic and evolutionary implications of a diverse paleobiota in amber from the early Eocene of India. *Proc. Natl. Acad. Sci.* **107**, 18360–18365 (2010).

94.	A. A. Agudelo, A new genus and species of Mantoididae (Mantodea) from the Brazilian and Venezuelan Amazon, with remarks on Mantoida Newman, 1838. *Zootaxa* **3797**, 194–206 (2014).

95.	T. Schubnel, A. Nel, New Paleogene mantises from the Oise amber and their evolutionary importance. *Acta Palaeontol. Pol.* **64** (2019).

96.	J. Szwedo, The Coleorrhyncha (Insecta: Hemiptera) of the European Jurassic, with a description of a new genus from the Toarcian of Luxembourg. *Vol. Jurassica* **9**, 3–20 (2011).

97.	D. Burckhardt, Taxonomy and phylogeny of the Gondwanan moss bugs or Peloridiidae (Hemiptera, Coleorrhyncha). *Dtsch. Entomol. Z.* **56**, 173–235 (2009).

98.	D. J. Field, Big-time insights from a tiny bird fossil. *Proc. Natl. Acad. Sci.* **114**, 7750–7752 (2017).

99.	D. T. Ksepka, T. A. Stidham, T. E. Williamson, Early Paleocene landbird supports rapid phylogenetic and morphological diversification of crown birds after the K–Pg mass extinction. *Proc. Natl. Acad. Sci.* **114**, 8047–8052 (2017).

100.	D. T. Ksepka, J. A. Clarke, Affinities of Palaeospiza bella and the phylogeny and biogeography of Mousebirds (Coliiformes). *The Auk* **126**, 245–259 (2009).

101.	N. V. Zelenkov, G. J. Dyke, The fossil record and evolution of mousebirds (aves: Coliiformes). *Palaeontology* **51**, 1403–1418 (2008).

102.	F. L. Agnolín, New fossil birds from the miocene of Patagonia, Argentina. *Poeyana* (2022).

103.	J. A. Clarke, D. T. Ksepka, N. A. Smith, M. A. Norell, Combined phylogenetic analysis of a new North American fossil species confirms widespread Eocene distribution for stem rollers (Aves, Coracii). *Zool. J. Linn. Soc.* **157**, 586–611 (2009).

104.	F. J. Degrange, D. Pol, P. Puerta, P. Wilf, Unexpected larger distribution of paleogene stem-rollers (Aves, Coracii): new evidence from the Eocene of Patagonia, Argentina. *Sci. Rep.* **11**, 1363 (2021).

105.	J. L. Conrad, A new Eocene casquehead lizard (Reptilia, Corytophanidae) from North America. *PLOS ONE* **10**, e0127900 (2015).

106.	K. T. Smith, A new lizard assemblage from the earliest eocene (Zone Wa0) of the bighorn basin, wyoming, USA: Biogeography during the warmest interval of the cenozoic. *J. Syst. Palaeontol.* **7**, 299–358 (2009).

107.	J. J. Eberle, M. D. Gottfried, J. H. Hutchison, C. A. Brochu, First record of Eocene bony fishes and crocodyliforms from Canada’s Western Arctic. *PLOS ONE* **9**, e96079 (2014).

108.	K. F. Hirsch, Fossil crocodilian eggs from the Eocene of Colorado. *J. Paleontol.* **59**, 531–542 (1985).

109.	I. T. Kuzmin, E. A. Zvonok, Crocodylian assemblage from the middle Eocene Ikovo locality (Lugansk Province, Ukraine), with a discussion of the fossil record and geographic origins of crocodyliform fauna in the Paleogene of Europe. *Geobios* **65**, 7–27 (2021).

110.	K. T. Lindblad, J. W. Moreno-Bernal, R. C. McKellar, M. I. Velez, The Northern Crocodile: first report of Borealosuchus (Eusuchia; Crocodylia) from Saskatchewan’s lower Ravenscrag Formation (earliest Paleocene) with implications for biogeography. *Can. J. Earth Sci.* **59**, 623–638 (2022).

111.	P. J. Markwick, Crocodilian diversity in space and time: the role of climate in paleoecology and its implication for understanding K/T extinctions. *Paleobiology* **24**, 470–497 (1998).

112.	P. J. Markwick, Fossil crocodilians as indicators of Late Cretaceous and Cenozoic climates: implications for using palaeontological data in reconstructing palaeoclimate. *Palaeogeogr. Palaeoclimatol. Palaeoecol.* **137**, 205–271 (1998).

113.	D. Tyborowski, B. Błażejowski, New marine reptile fossils from the Late Jurassic of Poland with implications for vertebrate faunas palaeobiogeography. *Proc. Geol. Assoc.* **130**, 741–751 (2019).

114.	N. Kobatake, *et al.*, Discovery of Crocodile Fossil from the Ôsaka Group. *Quat. Res. Daiyonki-Kenkyu* **4**, 49-58_2 (1965).

115.	R. López-Antoñanzas, F. and Knoll, A comprehensive phylogeny of the gundis (Ctenodactylinae, Ctenodactylidae, Rodentia). *J. Syst. Palaeontol.* **9**, 379–398 (2011).

116.	R. López-Antoñanzas, V. Gutkin, R. Rabinovich, R. Calvo, A. Grossman, A Transitional Gundi (Rodentia: Ctenodactylidae) from the Miocene of Israel. *PLOS ONE* **11**, e0151804 (2016).

117.	A. Oliver Pérez, G. Daxner-Höck, Large-sized species of ctenodactylidae from the Valley of Lakes (Mongolia): An update on dental morphology, biostratigraphy, and paleobiogeography. (2017).

118.	M. Vianey-Liaud, H. Gomes Rodrigues, L. Marivaux, A new Oligocene Ctenodactylinae (Rodentia: Mammalia) from Ulantatal (nei Mongol): new insight on the phylogenetic origins of the modern Ctenodactylidae. *Zool. J. Linn. Soc.* **160**, 531–550 (2010).

119.	R. N. Vasishat, R. Gaur, S. R. K. Chopra, Community structure of Middle Śivalik vertebrates from haritalyangar (H.P.), India. *Palaeogeogr. Palaeoclimatol. Palaeoecol.* **23**, 131–140 (1978).

120.	R. López-Antoñanzas, F. Knoll, S. Maksoud, D. Azar, First Miocene rodent from Lebanon provides the “missing link” between Asian and African gundis (Rodentia: Ctenodactylidae). *Sci. Rep.* **5**, 12871 (2015).

121.	R. L. Antoñanzas, S. Sen, Ctenodactylids from the Lower and Middle Miocene of Saudi Arabia. *Palaeontology* **47**, 1477–1494 (2004).

122.	M. Coiro, R. Allio, N. Mazet, L. J. Seyfullah, F. L. Condamine, Reconciling fossils with phylogenies reveals the origin and macroevolutionary processes explaining the global cycad biodiversity. *New Phytol.* **240**, 1616–1635 (2023).

123.	B. Erdei, *et al.*, First cycad seedling foliage from the fossil record and inferences for the Cenozoic evolution of cycads. *Biol. Lett.* **15**, 20190114 (2019).

124.	M. Coiro, C. Pott, Eobowenia gen. nov. from the Early Cretaceous of Patagonia: indication for an early divergence of Bowenia? *BMC Evol. Biol.* **17**, 97 (2017).

125.	B. Erdei, S. R. Manchester, Z. Kvaček, Dioonopsis Horiuchi et Kimura leaves from the Eocene of Western North America: a cycad shared with the Paleogene of Japan. *Int. J. Plant Sci.* **173**, 81–95 (2012).

126.	B. Erdei, S. R. Manchester, Ctenis clarnoensis sp. n., an unusual cycadalean foliage from the Eocene Clarno Formation, Oregon. *Int. J. Plant Sci.* **176**, 31–43 (2015).

127.	J. Horiuchi, T. Kimura, Dioonopsis nipponica gen. et. sp. nov., a new cycad from the palaeogene of Japan. *Rev. Palaeobot. Palynol.* **51**, 213–225 (1987).

128.	K. Uzunova, E. Palamarev, Z. Kvacek, Eostangeria ruzinciniana (Zamiaceae) from the Middle Miocene of Bulgaria and its relationship to similar taxa of fossil Eostangeria, and extant Chigua and Stangeria (Cycadales). *Acta Palaeobot.* **41** (2001).

129.	K. Su, C. Quan, Y.-S. (Christopher) Liu, Cycas fushunensis sp. nov. (Cycadaceae) from the Eocene of northeast China. *Rev. Palaeobot. Palynol.* **204**, 43–49 (2014).

130.	B. Erdei, F. Akgün, M. R. Barone Lumaga, Pseudodioon akyoli gen. et sp. nov., an extinct member of Cycadales from the Turkish Miocene. *Plant Syst. Evol.* **285**, 33–49 (2010).

131.	C. Pott, H. Kerp, M. Krings, Pseudoctenis cornelii nov. spec. (cycadalean foliage) from the Carnian (Upper Triassic) of Lunz, Lower Austria. *Ann. Naturhistorischen Mus. Wien Ser. Für Mineral. Petrogr. Geol. Paläontol. Anthropol. Prähistorie* **109**, 1–17 (2007).

132.	Z. Kvaček, S. R. Manchester, Eostangeria Barthel (Extinct Cycadales) from the Paleogene of Western North America and Europe. *Int. J. Plant Sci.* **160**, 621–629 (1999).

133.	H. G. McDonald, S. F. Vizcaíno, S. Bargo, “Skeletal anatomy and the fossil historyof the Vermilingua” in *The Biology of the Xenarthra*, S. F. Vizcaíno, W. J. Loughry, Eds. (University Press of Florida, 2008), pp. 64–78.

134.	M. G. Vucetich, D. H. Verzi, First record of Dasyproctidae (Rodentia) in the Pleistocene of Argentina. Paleoclimatic implication. *Palaeogeogr. Palaeoclimatol. Palaeoecol.* **178**, 67–73 (2002).

135.	J. Jenkins Shaw, E. Perkovsky, A. Ślipiński, H. Escalona, A. Solodovnikov, An extralimital fossil of the genus Diagrypnodes (Coleoptera: Salpingidae: Inopeplinae). *Hist. Biol.* **36**, 1196–1203 (2024).

136.	M. R. Dawson, L. Marivaux, C. Li, K. C. Beard, G. Métais, Laonastes and the “Lazarus Effect” in recent mammals. *Science* **311**, 1456–1458 (2006).

137.	L. J. Flynn, Origin and evolution of the Diatomyidae, with clues to Paleoecology from the fossil record. *Bull. Carnegie Mus. Nat. Hist.* **2007**, 173–181 (2007).

138.	L. J. Flynn, M. E. Morgan, An unusual diatomyid rodent from an infrequently sampled late Miocene interval in the Siwaliks of Pakistan. *Palaeontol. Electron.* **8**, 17A (2005).

139.	R. López-Antoñanzas, First diatomyid rodent from the Early Miocene of Arabia. *Naturwissenschaften* **98**, 117–123 (2011).

140.	L. Marivaux, J.-L. Welcomme, New diatomyid and baluchimyine rodents from the Oligocene of Pakistan (Bugti Hills, Balochistan): systematic and paleobiogeographic implications. *J. Vertebr. Paleontol.* **23**, 420–434 (2003).

141.	Z. Marković, W. Wessels, A. A. van de Weerd, H. de Bruijn, On a new diatomyid (Rodentia, Mammalia) from the Paleogene of south-east Serbia, the first record of the family in Europe. *Palaeobiodiversity Palaeoenvironments* **98**, 459–469 (2018).

142.	A. M. Lister, A. V. Sher, H. van Essen, G. Wei, The pattern and process of mammoth evolution in Eurasia. *Quat. Int.* **126–128**, 49–64 (2005).

143.	A. M. Lister, A. V. Sher, Evolution and dispersal of mammoths across the Northern Hemisphere. *Science* **350**, 805–809 (2015).

144.	D. Nogués-Bravo, J. Rodríguez, J. Hortal, P. Batra, M. B. Araújo, Climate Change, Humans, and the Extinction of the Woolly Mammoth. *PLOS Biol.* **6**, e79 (2008).

145.	A. Soszyńska-Maj, W. Krzemiński, K. Kopeć, R. A. Coram, Phylogenetic relationships within the relict family Eomeropidae (Insecta, Mecoptera) based on the oldest fossil from the Early Jurassic (Sinemurian) of Dorset, southern England. *J. Syst. Palaeontol.* **14**, 1025–1031 (2016).

146.	B. Wang, *et al.*, The mid-Miocene Zhangpu biota reveals an outstandingly rich rainforest biome in East Asia. *Sci. Adv.* **7**, eabg0625 (2021).

147.	S. B. Archibald, A. P. Rasnitsyn, Two new species of fossil Eomerope (Mecoptera: Eomeropidae) from the Ypresian Okanagan Highlands, far-western North America, and Eocene Holarctic dispersal of the genus. *Can. Entomol.* **150**, 393–403 (2018).

148.	S. B. Archibald, A. P. Rasnitsyn, M. A. Akhmetiev, Ecology and distribution of Cenozoic Eomeropidae (Mecoptera), and a new species of Eomerope Cockerell from the Early Eocene McAbee locality, British Columbia, Canada. *Ann. Entomol. Soc. Am.* **98**, 503–514 (2005).

149.	J. Zhang, C. Shih, J. F. Petrulevičius, D. Ren, A new fossil eomeropid (Insecta, Mecoptera) from the Jiulongshan Formation, Inner Mongolia, China. *Zoosystema* **33**, 443–450 (2011).

150.	X. Zhao, X. Zhao, L. Chen, Q. Zhang, B. Wang, A new species of Eomeropidae (Insecta: Mecoptera) from the Middle Jurassic of China. *Proc. Geol. Assoc.* **130**, 691–695 (2019).

151.	H. Wang, Z. Yao, J. Wang, Q. Li, J. Yang, The first discovery of Eomeropidae (Insecta: Mecoptera) from the Lower Jurassic of northwestern China. *Hist. Biol.* 1–5 (2023). https://doi.org/10.1080/08912963.2023.2250821.

152.	O. Cirilli, *et al.*, Evolution of the Family Equidae, Subfamily Equinae, in North, Central and South America, Eurasia and Africa during the Plio-Pleistocene. *Biology* **11**, 1258 (2022).

153.	M. O. Woodburne, B. J. MacFadden, A reappraisal of the systematics, biogeography, and evolution of fossil horses. *Paleobiology* **8**, 315–327 (1982).

154.	A. M. Candela, M. G. Vucetich, Hypsosteiromys (Rodentia, Hystricognathi) from the Early Miocene of Patagonia (Argentina), the only Erethizontidae with a tendency to hypsodonty. *Geobios* **35**, 153–161 (2002).

155.	A. M. Candela, A new giant porcupine (Rodentia, Erethizontidae) from the late Miocene of Argentina. *J. Vertebr. Paleontol.* **24**, 732–741 (2004).

156.	D. R. Sussman, The erethizontid fossil from the Uquía formation of Argentina should not be referred to the genus *Erethizon*. *J. South Am. Earth Sci.* **31**, 475–478 (2011).

157.	R. I. Vezzosi, L. Kerber, The southernmost record of a large erethizontid rodent (Hystricomorpha: Erethizontoidea) in the Pleistocene of South America: Biogeographic and paleoenvironmental implications. *J. South Am. Earth Sci.* **82**, 76–90 (2018).

158.	J. Heinrichs, A. Schäfer-Verwimp, K. Feldberg, A. R. Schmidt, The extant liverwort Gackstroemia (Lepidolaenaceae, Porellales) in Cretaceous amber from Myanmar. *Rev. Palaeobot. Palynol.* **203**, 48–52 (2014).

159.	D. L. Royer, L. J. Hickey, S. L. Wing, Ecological conservatism in the “living fossil” Ginkgo. *Paleobiology* **29**, 84–104 (2003).

160.	G. Iliopoulos, S. Roussiakis, “The Fossil Record of Giraffes (Mammalia: Giraffidae) in Greece” in *Fossil Vertebrates of Greece Vol. 2: Laurasiatherians, Artiodactyles, Perissodactyles, Carnivorans, and Island Endemics*, E. Vlachos, Ed. (Springer International Publishing, 2022), pp. 301–333.

161.	K. Laskos, D. S. Kostopoulos, On the last European giraffe, Palaeotragus inexspectatus (Mammalia: Giraffidae); new remains from the Early Pleistocene of Greece and a review of the species. *Zool. J. Linn. Soc.* **203**, zlae056 (2025).

162.	M. Ríos, I. M. Sánchez, J. Morales, A new giraffid (Mammalia, Ruminantia, Pecora) from the late Miocene of Spain, and the evolution of the sivathere-samothere lineage. *PLoS ONE* **12**, e0185378 (2017).

163.	S. Wedmann, M. Poschmann, T. Hörnschemeyer, Fossil insects from the Late Oligocene Enspel Lagerstätte and their palaeobiogeographic and palaeoclimatic significance. *Palaeobiodiversity Palaeoenvironments* **90**, 49–58 (2010).

164.	J. I. Noriega, F. Agnolin, El registro paleontológico de las Aves del “Mesopotamiense” (Formación Ituzaingó; Mioceno tardío-Plioceno) de la provincia de Entre Ríos, Argentina. (2008).

165.	X. Xiang, K. Xiang, R. D. C. Ortiz, F. Jabbour, W. Wang, Integrating palaeontological and molecular data uncovers multiple ancient and recent dispersals in the pantropical Hamamelidaceae. *J. Biogeogr.* **46**, 2622–2631 (2019).

166.	P. K. Endress, E. M. Friis, Archamamelis, hamamelidalean flowers from the Upper Cretaceous of Sweden. *Plant Syst. Evol.* **175**, 101–114 (1991).

167.	M. Collinson, S. Manchester, V. Wilde, Fossil fruits and seeds of the Middle Eocene Messel biota, Germany. *Abh. Senckenberg Ges. Für Naturforschung* **570**, 1–250 (2012).

168.	M. G. Radtke, K. B. Pigg, W. C. Wehr, Fossil Corylopsis and Fothergilla leaves (Hamamelidaceae) from the Lower Eocene flora of Republic, Washington, U.S.A., and their evolutionary and biogeographic significance. *Int. J. Plant Sci.* **166**, 347–356 (2005).

169.	T. Tanai, On the Hamamelidaceae from the Palaeogene of Hokkaido, Japan. *Trans. Proc. Palaeontol. Soc. Jpn.* **66**, 56–62 (1967).

170.	N. F. Adams, I. Candy, D. C. Schreve, An Early Pleistocene hippopotamus from Westbury Cave, Somerset, England: support for a previously unrecognized temperate interval in the British Quaternary record. *J. Quat. Sci.* **37**, 28–41 (2022).

171.	D. Fidalgo, J. Madurell-Malapeira, R. Martino, L. Pandolfi, A. Rosas, An Updated Review of The Quaternary Hippopotamus Fossil Records from the Iberian Peninsula. *Quaternary* **7**, 4 (2024).

172.	D. Penney, P. A. Selden, First fossil Huttoniidae (Arthropoda: Chelicerata: Araneae) in late Cretaceous Canadian amber. *Cretac. Res.* **27**, 442–446 (2006).

173.	N. Rohland, *et al.*, The Population History of Extant and Extinct Hyenas. *Mol. Biol. Evol.* **22**, 2435–2443 (2005).

174.	J. L. Prado, B. Azanza, J. L. Cantalapiedra, M. T. Alberdi, Plio-Pleistocene fossil record of large predators in Iberia: Diversity, home range and climatic change. *Palaeogeogr. Palaeoclimatol. Palaeoecol.* **399**, 404–413 (2014).

175.	L. Rinehart, S. Lucas, “A population of Antediplodon dockumensis (Bivalvia : Unionoida : Hyriidae) from the Revueltian (Upper Triassic : Early-Mid Norian) Bull Canyon Formation of West Texas: growth, allometry, productivity, and ecology” in *The Triassic System: New Developments in Stratigraphy and Paleontology*, Bulletin of the New Mexico Museum of Natural History and Science., (2013), pp. 500–523.

176.	J. F. McAlpine, A fossil Ironomyiid fly from Canadian amber (Diptera:Ironomyiidae). *Can. Entomol.* **105**, 105–111 (1973).

177.	X. Li, D. K. Yeates, The first Ironomyiidae from mid-Cretaceous Burmese amber provides insights into the phylogeny of Phoroidea (Diptera: Cyclorrhapha). *Syst. Entomol.* **44**, 251–261 (2019).

178.	E. J. Hermsen, A review of the fossil record of the genus Itea (Iteaceae, Saxifragales) with comments on its historical biogeography. *Bot. Rev.* **79**, 1–47 (2013).

179.	Y.-M. Tian, J. Huang, T. Su, S.-T. Zhang, Early Oligocene Itea (Iteaceae) leaves from East Asia and their biogeographic implications. *Plant Divers.* **43**, 142–151 (2021).

180.	E. Martinetto, The role of central Italy as a centre of refuge for thermophilous plants in the late Cenozoic. *Acta Palaeobot.* **41**, 299–319 (2001).

181.	P. I. Dorofeev, *Miocenovye flory Tambovskoj oblasti* (Nauka, 1988).

182.	E. Palamarev, V. Bozukov, K. Uzunova, A. Petkova, G. Kitanov, Catalogue of the Cenozoic plants of Bulgaria (Eocene to Pliocene). *Phitologia Balc.* **11**, 2005 (2005).

183.	R. Y. Smith, J. F. Basinger, D. R. Greenwood, Depositional setting, fossil flora, and paleoenvironment of the Early Eocene Falkland site, Okanagan Highlands, British Columbia. *Can. J. Earth Sci.* **46**, 811–822 (2009).

184.	P. T. Moss, D. R. Greenwood, S. B. Archibald, Regional and local vegetation community dynamics of the Eocene Okanagan Highlands (British Columbia  Washington State) from palynology. *Can. J. Earth Sci.* **42**, 187–204 (2005).

185.	C. Gruas-Cavagnetto, *Étude palynologique du paléogène du sud de l’Angleterre* (Centre national de la recherche scientifique, 1976).

186.	M. Poschmann, T. Schindler, D. Uhl, Fossil-Lagerstätte Enspel–a short review of current knowledge, the fossil association, and a bibliography. *Palaeobiodiversity Palaeoenvironments* **90**, 3–20 (2010).

187.	V. Bozukov, T. Utescher, D. Ivanov, Late Eocene to early Miocene climate and vegetation of Bulgaria. *Rev. Palaeobot. Palynol.* **153**, 360–374 (2009).

188.	A. Traverse, Palynofloral geochronology of the Brandon Lignite of Vermont, USA. *Rev. Palaeobot. Palynol.* **82**, 265–297 (1994).

189.	M. Ziembinska-Tworzydlo, Palynological characteristics of the Neogene of Western Poland. *Acta Palaeontol. Pol.* **19** (1974).

190.	L. Seitner, Miocene microflora of Rittsteig (Germany). PANGAEA. https://doi.org/10.1594/PANGAEA.206682. Deposited 2004.

191.	P. Walsh, I. Morawiecka, K. Skawińska-Wieser, A Miocene palynoflora preserved by karstic subsidence in Anglesey and the origin of the Menaian Surface. *Geol. Mag.* **133**, 713–719 (1996).

192.	D. Ivanov, A. R. Ashraf, V. Mosbrugger, E. Palamarev, Palynological evidence for Miocene climate change in the Forecarpathian Basin (Central Paratethys, NW Bulgaria). *Palaeogeogr. Palaeoclimatol. Palaeoecol.* **178**, 19–37 (2002).

193.	M. Kayseri, F. Akgün, Palynostratigraphic, palaeovegetational and palaeoclimatic investigations on the Miocene deposits of central Anatolia (Çorum Region and Sivas Basin). *Turk. J. Earth Sci.* **17**, 361–403 (2008).

194.	E. Worobiec, Middle Miocene palynoflora of the Legnica lignite deposit complex, Lower Silesia, Poland. *Acta Palaeobot.* **49**, 5–133 (2009).

195.	D. K. Ferguson, M. Pingen, R. Zetter, C.-C. Hofmann, Advances in our knowledge of the Miocene plant assemblage from Kreuzau, Germany. *Rev. Palaeobot. Palynol.* **101**, 147–177 (1998).

196.	L. Seitner, Miocene microflora of Achldorf (Germany). PANGAEA. https://doi.org/10.1594/PANGAEA.206519. Deposited 2004.

197.	C.-C. Hofmann, R. Zetter, Reconstruction of different wetland plant habitats of the Pannonian Basin system (Neogene, Eastern Austria). *PALAIOS* **20**, 266–279 (2005).

198.	A. Bertini, E. Martinetto, Reconstruction of vegetation transects for the Messinian–Piacenzian of Italy by means of comparative analysis of pollen, leaf and carpological records. *Palaeogeogr. Palaeoclimatol. Palaeoecol.* **304**, 230–246 (2011).

199.	A. Bertini, The Northern Apennines palynological record as a contribute for the reconstruction of the Messinian palaeoenvironments. *Sediment. Geol.* **188–189**, 235–258 (2006).

200.	S.-M. Popescu, *et al.*, Pliocene and Lower Pleistocene vegetation and climate changes at the European scale: Long pollen records and climatostratigraphy. *Quat. Int.* **219**, 152–167 (2010).

201.	P. M. Brito, J. Alvarado-Ortega, F. J. Meunier, Earliest known lepisosteoid extends the range of anatomically modern gars to the Late Jurassic. *Sci. Rep.* **7**, 17830 (2017).

202.	C. D. Brownstein, L. Yang, M. Friedman, T. J. Near, Phylogenomics of the Ancient and Species-Depauperate Gars Tracks 150 Million Years of Continental Fragmentation in the Northern Hemisphere. *Syst. Biol.* **72**, 213–227 (2023).

203.	S. L. A. Cooper, J. Gunn, P. M. Brito, S. Zouhri, D. M. Martill, A new fully marine, short-snouted lepisosteid gar from the Upper Cretaceous (Turonian) of North Africa. *Cretac. Res.* **151**, 105650 (2023).

204.	M. D. Gottfried, D. W. Krause, First record of gars (Lepisosteidae, Actinopterygii) on Madagascar: Late Cretaceous remains from the Mahajanga Basin. *J. Vertebr. Paleontol.* **18**, 275–279 (1998).

205.	V. Nikolov, P. Andreev, G. Voykov, D. Dochev, The first record of gars (Ginglymodi: Lepisosteidae) from the Upper Cretaceous of Bulgaria reveals a wider paleogeographic distribution of lepisosteids within the European Archipelago. *Cretac. Res.* **165**, 105985 (2025).

206.	M. Szabó, P. Gulyás, A. Ősi, Late Cretaceous (Santonian) Atractosteus (Actinopterygii, Lepisosteidae) remains from Hungary (Iharkút, Bakony Mountains). *Cretac. Res.* **60**, 239–252 (2016).

207.	F. Ortega, *et al.*, The biota of the Upper Cretaceous site of Lo Hueco (Cuenca, Spain). *J. Iber. Geol.* **41**, 83–99 (2015).

208.	V. Codrea, A. Solomon, Peculiar fossilization and taphonomy in Maastrichtian terrestrial deposits of Pui (Haţeg Basin, Romania). Studii şi cercetǎri. *Stud. Si Cercet. Geol.-Geogr.* **17**, 51–69 (2012).

209.	A. Blanco, M. Szabó, À. Blanco-Lapaz, J. Marmi, Late Cretaceous (Maastrichtian) Chondrichthyes and Osteichthyes from northeastern Iberia. *Palaeogeogr. Palaeoclimatol. Palaeoecol.* **465**, 278–294 (2017).

210.	P. M. Brito, A. E. Piacentini Pinheiro, T. B. Ribeiro, M. A. Medeiros, Lepisosteidae remains (Holostei: Ginglymodi) from the Middle Cretaceous Alcântara Formation, Northeastern Brazil. *J. South Am. Earth Sci.* 105289 (2024). https://doi.org/10.1016/j.jsames.2024.105289.

211.	P. Barden, B. Boudinot, A. Lucky, Where fossils dare and males matter: combined morphological and molecular analysis untangles the evolutionary history of the spider ant genus Leptomyrmex Mayr (Hymenoptera : Dolichoderinae). *Invertebr. Syst.* **31**, 765–780 (2017).

212.	B. E. Boudinot, R. S. Probst, C. R. F. Brandão, R. M. Feitosa, P. S. Ward, Out of the Neotropics: newly discovered relictual species sheds light on the biogeographical history of spider ants (Leptomyrmex, Dolichoderinae, Formicidae). *Syst. Entomol.* **41**, 658–671 (2016).

213.	D. J. Field, A. Y. Hsiang, A North American stem turaco, and the complex biogeographic history of modern birds. *BMC Evol. Biol.* **18**, 102 (2018).

214.	G. Mayr, The Madagascan “Cuckoo-roller” (Aves: Leptosomidae) is not a roller — notes on the phylogenetic affinities and evolutionary history of a “living fossil.” *Acta Ornithol.* **43**, 226–230 (2008).

215.	I. Weidig, The first New World occurrence of the Eocene birdPlesiocathartes (Aves: ?Leptosomidae). *Paläontol. Z.* **80**, 230–237 (2006).

216.	J. Szwedo, A. Stroiński, Q. Lin, Tip of the clade on the top of the World—the first fossil Lophopidae (Hemiptera: Fulgoromorpha) from the Palaeocene of Tibet. *Sci. Nat.* **102**, 28 (2015).

217.	A. F. Fernández-Aldea, K. R. Barão, J. Grazia, A. Ferrari, An Integrative Approach to the Taxonomy of Oenopiella Bergroth (Hemiptera: Heteroptera: Pentatomidae: Pentatominae: Carpocorini) with the Description of Two New Species from Argentina and Southern Brazil. *Ann. Entomol. Soc. Am.* **107**, 364–381 (2014).

218.	J. J. Hooker, D. E. Russell, Early Palaeogene Louisinidae (Macroscelidea, Mammalia), their relationships and north European diversity. *Zool. J. Linn. Soc.* **164**, 856–936 (2012).

219.	C. C. Davis, P. W. Fritsch, C. D. Bell, S. Mathews, High‐latitude Tertiary migrations of an exclusively tropical clade: evidence from Malpighiaceae. *Int. J. Plant Sci.* **165**, S107–S121 (2004).

220.	C. C. Davis, C. D. Bell, S. Mathews, M. J. Donoghue, Laurasian migration explains Gondwanan disjunctions: Evidence from Malpighiaceae. *Proc. Natl. Acad. Sci.* **99**, 6833–6837 (2002).

221.	L. Hably, S. R. Manchester, Fruits of Tetrapterys (Malpighiaceae) from the Oligocene of Hungary and Slovenia. *Rev. Palaeobot. Palynol.* **111**, 93–101 (2000).

222.	D. W. Taylor, W. L. Crepet, Fossil floral evidence of Malpighiaceae and an early plant-pollinator relationship. *Am. J. Bot.* **74**, 274–286 (1987).

223.	E. Conti, C. Mulder, F. Lombardo, A. Marletta, G. Costa, The present is the key to the past: how living fossils in Namibia share insights on the insects of Tertiary European forests. *Afr. Entomol.* **27**, 185–200 (2019).

224.	M. J. B. Eberhard, M. D. Picker, K.-D. Klass, Sympatry in Mantophasmatodea, with the description of a new species and phylogenetic considerations. *Org. Divers. Evol.* **11**, 43–59 (2011).

225.	B. Wipfler, T. Theska, R. Predel, Mantophasmatodea from the Richtersveld in South Africa with description of two new genera and species. *ZooKeys* 137–160 (2018). https://doi.org/10.3897/zookeys.746.14885.

226.	B. Wipfler, H. Pohl, R. Predel, Two new genera and two new species of Mantophasmatodea (Insecta, Polyneoptera) from Namibia. *ZooKeys* **166**, 75–98 (2012).

227.	O. Zompro, J. Adis, Notes on Namibian Mantophasma Zompro, Klass, Kristensen & Adis, 2002, with descriptions of three new species (Insecta: Mantophasmatodea: Mantophasmatidae: Mantophasmatini). *Russ. Entomol. J.* **15**, 21–24 (2006).

228.	M. S. Engel, E. D. Currano, B. F. Jacobs, The first mastotermitid termite from Africa (Isoptera: Mastotermitidae): a new species of Mastotermes from the early Miocene of Ethiopia. *J. Paleontol.* **89**, 1038–1042 (2015).

229.	Z. Zhao, *et al.*, The oldest known mastotermitids (Blattodea: Termitoidae) and phylogeny of basal termites. *Syst. Entomol.* **44**, 612–623 (2019).

230.	T. Wappler, M. S. Engel, A new record of Mastotermes from the Eocene of Germany (Isoptera: Mastotermitidae). *J. Paleontol.* **80**, 380–385 (2006).

231.	P. Piñero, *et al.*, La Piquera in central Iberian Peninsula: A new key vertebrate locality for the Early Pliocene of western Europe. (2023).

232.	L. Vilhelmsen, V. Perrichot, S. R. Shaw, Past and present diversity and distribution in the parasitic wasp family Megalyridae (Hymenoptera). *Syst. Entomol.* **35**, 658–677 (2010).

233.	C. O. Azevedo, M. T. Tavares, A new species of Cryptalyra (Hymenoptera, Megalyridae) from Brazil with a key to species of the genus. *Zootaxa* **1259**, 55–59 (2006).

234.	F. J. Goin, *et al.*, Nuevo marsupial (Mammalia) del Eoceno de la Antártida, y los orígenes y afinidades de los Microbiotheria. *Rev. Asoc. Geológica Argent.* **62**, 597–603 (2007).

235.	S. B. Archibald, S. P. Cover, C. S. Moreau, Bulldog ants of the Eocene Okanagan Highlands and history of the subfamily (Hymenoptera: Formicidae: Myrmeciinae). *Ann. Entomol. Soc. Am.* **99**, 487–523 (2006).

236.	G. M. Dlussky, New fossil ants of the subfamily Myrmeciinae (Hymenoptera, Formicidae) from Germany. *Paleontol. J.* **46**, 288–292 (2012).

237.	C. Jouault, A. Nel, The oldest Cenozoic ant fossil: Tyrannomecia gen. nov. (Formicidae: Myrmeciinae) from the Palaeocene Menat Formation (France). *Hist. Biol.* **34**, 2241–2248 (2022).

238.	G. M. Dlussky, A. P. Rasnitsyn, P. K.S, The ants (Hymenoptera: Formicidae) of Bol’shaya Svetlovodnaya (Late Eocene of Sikhote-Alin, Russian Far East). *Cauc. Entomol. Bull.* **11**, 131–152 (2015).

239.	A. G. Radchenko, E. E. Perkovsky, New finds of the fossil ant genus Prionomyrmex Mayr (Hymenoptera, Formicidae, Myrmeciinae) in Late Eocene European amber. *Paleontol. J.* **54**, 617–626 (2020).

240.	G. F. Gunnell, N. B. Simmons, E. R. Seiffert, New Myzopodidae (Chiroptera) from the Late Paleogene of Egypt: Emended Family Diagnosis and Biogeographic Origins of Noctilionoidea. *PLOS ONE* **9**, e86712 (2014).

241.	Y. Cao, X. Lin, C. Shih, D. Ren, Two new species of Itaphlebia (Insecta, Mecoptera, Nannochoristidae) from the late Middle Jurassic of China. *ZooKeys* **1108**, 175–188 (2022).

242.	N. Liu, Y. Zhao, D. Ren, Two new fossil species of Itaphlebia (Mecoptera: Nannochoristidae) from Jiulongshan Formation, Inner Mongolia, China. *Zootaxa* **2420**, 37–45 (2010).

243.	F. Bibi, A. Hill, M. Beech, W. Yasin, “Late Miocene Fossils from the Baynunah Formation, United Arab Emirates: Summary of a Decade of New Work” in *Fossil Mammals of Asia: Neogene Biostratigraphy and Chronology*, M. Fortelius, X. Wang, L. Flynn, Eds. (Columbia University Press, 2013), p. 0.

244.	B. Kraatz, “Rodents from the Baynunah Formation” in *Sands of Time: Ancient Life in the Late Miocene of Abu Dhabi, United Arab Emirates*, F. Bibi, B. Kraatz, M. J. Beech, A. Hill, Eds. (Springer International Publishing, 2022), pp. 191–201.

245.	A. García-Alix, *et al.*, Updating the Europe–Africa small mammal exchange during the late Messinian. *J. Biogeogr.* **43**, 1336–1348 (2016).

246.	L. A. Lavrenchenko, R. S. Nadjafova, A. Bekele, T. A. Mironova, J. Bryja, Phylogenetic position of a monotypic Ethiopian endemic rodent genus Megadendromus (Rodentia, Nesomyidae). *Mammalia* **81**, 71–82 (2017).

247.	Y. Cui, D. Ren, O. Béthoux, The Pangean journey of ‘south forestflies’ (Insecta: Plecoptera) revealed by their first fossils. *J. Syst. Palaeontol.* **17**, 255–268 (2019).

248.	I. D. McLellan, Six new species and a new genus of stoneflies (Plecoptera) from New Zealand. *N. Z. J. Zool.* **30**, 101–113 (2003).

249.	G. Mayr, The Palaeogene Old World potoo Paraprefica Mayr, 1999 (Aves, Nyctibiidae): Its osteology and affinities to the New World Preficinae Olson, 1987. *J. Syst. Palaeontol.* **3**, 359–370 (2005).

250.	T. V. V. Costa, L. F. Silveira, S. R. Posso, R. J. Donatelli, Phylogenetic analysis of the nocturnal avian family Nyctibiidae (Caprimulgiformes) inferred from osteological characters. *Zool. Anz.* **291**, 113–122 (2021).

251.	C. Mourer-Chauviré, New data concerning the familial position of the genus Euronyctibius (Aves, Caprimulgiformes) from the paleogene of the Phosphorites du Quercy, France. *Paleontol. J.* **47**, 1315–1322 (2013).

252.	S. B. Archibald, V. N. Makarkin, A new genus and species of split-footed lacewings (Neuroptera) from the early Eocene of western Canada and revision of the subfamily affinities of Mesozoic Nymphidae. *Can. Entomol.* **152**, 269–287 (2020).

253.	S. B. Archibald, V. N. Makarkin, J. Ansorge, New fossil species of Nymphidae (Neuroptera) from the Eocene of North America and Europe. *Zootaxa* **2157** (2009).

254.	M. I. El-Soughier, R. C. Mehrotra, Z.-Y. Zhou, G.-L. Shi, *Nypa* fruits and seeds from the Maastrichtian–Danian sediments of Bir Abu Minqar, South Western Desert, Egypt. *Palaeoworld* **20**, 75–83 (2011).

255.	C. Gomez-Navarro, C. Jaramillo, F. Herrera, S. L. Wing, R. Callejas, Palms (Arecaceae) from a Paleocene rainforest of northern Colombia. *Am. J. Bot.* **96**, 1300–1312 (2009).

256.	D. M. Jarzen, D. L. Dilcher, Middle Eocene terrestrial palynomorphs from the Dolime Minerals and Gulf Hammock quarries, Florida, U.S.A. *Palynology* **30**, 89–110 (2006).

257.	O. Lenz, S. Diekmeier, V. Wilde, Nypa pollen diversity in middle Eocene mangroves at the edge of the southern Proto-North Sea (Helmstedt, northern Germany) – a statistical approach. *Grana* **60**, 97–118 (2021).

258.	J. Y. Lim, *et al.*, The Cenozoic history of palms: Global diversification, biogeography and the decline of megathermal forests. *Glob. Ecol. Biogeogr.* **31**, 425–439 (2022).

259.	J. Muller, Palynology of the Pedawan and Plateau Sandstone Formations (Cretaceous-Eocene) in Sarawak, Malaysia. *Micropaleontology* **14**, 1–37 (1968).

260.	D. T. Pocknall, C. D. Clowes, D. M. Jarzen, Spinizonocolpites prominatus (McIntyre) Stover & Evans: fossil Nypa pollen, taxonomy, morphology, global distribution, and paleoenvironmental significance. *N. Z. J. Geol. Geophys.* **66**, 558–570 (2023).

261.	D. T. Pocknall, R. N. Erlich, Palynostratigraphy and lithostratigraphy of Upper Cretaceous and Paleogene outcrop sections, Mérida Andes (Maracaibo Basin), Western Venezuela. *J. South Am. Earth Sci.* **104**, 102830 (2020).

262.	R. Zetter, C.-C. Hofmann, New aspects of the palynoflora of the lowermost Eocene (Krappfeld Area, Carinthia). *Österr. Akad. Wiss. Schriftenreihe Erdwissenschaftlichen Komm.* **14**, 473–507 (2001).

263.	S. Torricelli, G. Knezaurek, U. Biffi, Sequence biostratigraphy and paleoenvironmental reconstruction in the Early Eocene Figols Group of the Tremp–Graus Basin (south-central Pyrenees, Spain). *Palaeogeogr. Palaeoclimatol. Palaeoecol.* **232**, 1–35 (2006).

264.	W. Volkheimer, L. Scafati, D. Melendi, Palynology of a Danian warm climatic wetland in Central Northern Patagonia, Argentina. *Rev. Esp. Micropaleontol.* **39**, 117–134 (2007).

265.	W. Zhou, Q.-Y. (Jenny) Xiang, J. Wen, Phylogenomics, biogeography, and evolution of morphology and ecological niche of the eastern Asian–eastern North American Nyssa (Nyssaceae). *J. Syst. Evol.* **58**, 571–603 (2020).

266.	D. Ge, *et al.*, Did the expansion of C4 plants drive extinction and massive range contraction of micromammals? Inferences from food preference and historical biogeography of pikas. *Palaeogeogr. Palaeoclimatol. Palaeoecol.* **326–328**, 160–171 (2012).

267.	B. Moncunill-Solé, Eco-evolutionary adaptations of ochotonids (Mammalia: Lagomorpha) to islands: new insights into Late Miocene pikas from the Gargano palaeo-archipelago (Italy). *Biol. J. Linn. Soc.* **132**, 400–413 (2021).

268.	C. Laplana, *et al.*, How Far into Europe Did Pikas (Lagomorpha: Ochotonidae) Go during the Pleistocene? New Evidence from Central Iberia. *PLOS ONE* **10**, e0140513 (2015).

269.	Y.-D. Li, D.-Y. Huang, C.-Y. Cai, New species of Omma Newman from mid-Cretaceous Burmese amber (Coleoptera, Archostemata, Ommatidae). *Dtsch. Entomol. Z.* **68**, 341–348 (2021).

270.	J. Tan, Y. Wang, D. Ren, X. Yang, New fossil species of ommatids (Coleoptera: Archostemata) from the Middle Mesozoic of China illuminating the phylogeny of Ommatidae. *BMC Evol. Biol.* **12**, 113 (2012).

271.	A. G. Kirejtshuk, Taxonomic Review of Fossil Coleopterous Families (Insecta, Coleoptera). Suborder Archostemata: Superfamilies Coleopseoidea and Cupedoidea. *Geosciences* **10**, 73 (2020).

272.	S. B. Lee, G. S. Nam, J. K. Park, B. H. Lee, Y.-D. Li, Cretaceous beetles of the Jinju Formation (Coleoptera): Archostemata. *Palaeoentomology* **6**, 496–506 (2023).

273.	Z. Song, X. Zhao, E. A. Jarzembowski, C. Xiao, Overview of and taphonomic bias in Archostemata (Insecta: Coleoptera) from Cretaceous amber. *Palaeontogr. Abt. A* 119–137 (2022). https://doi.org/10.1127/pala/2022/0131.

274.	J. A. Dunlop, J. Wunderlich, G. O. P. Jr, The first fossil opilioacariform mite (Acari: Opilioacariformes) and the first Baltic amber camel spider (Solifugae). *Earth Environ. Sci. Trans. R. Soc. Edinb.* **94**, 261–273 (2003).

275.	N. P. I. Das, D. B. Bastawade, The first report of the acarine suborder Opilioacarida from India, with description of new genus, Indiacarus, and a new species, Indiacarus pratyushi. *Acarologia* **47**, 3–11 (2007).

276.	M. M. Vázquez, M. S. D. Araújo, R. J. F. Feres, Brasilacarus cocaris (Acari: Opilioacaridae), a new genus and species from Amazonia, Brazil. *Zootaxa* **3915**, 375–389 (2015).

277.	G. Mayr, V. L. De Pietri, Earliest and first Northern Hemispheric hoatzin fossils substantiate Old World origin of a “Neotropic endemic.” *Naturwissenschaften* **101**, 143–148 (2014).

278.	R. Pascual, *et al.*, First discovery of monotremes in South America. *Nature* **356**, 704–706 (1992).

279.	T. F. Flannery, *et al.*, A review of monotreme (Monotremata) evolution. *Alcheringa Australas. J. Palaeontol.* **46**, 3–20 (2022).

280.	V. M. Bravo-Cuevas, J. Priego-Vargas, M. Á. Cabral-Perdomo, M. A. Pineda Maldonado, First occurrence of *Panthera atrox* (Felidae, Pantherinae) in the Mexican state of Hidalgo and a review of the record of felids from the Pleistocene of Mexico. *Foss. Rec.* **19**, 131–141 (2016).

281.	D. W. G. Stanton, *et al.*, Early Pleistocene origin and extensive intra-species diversity of the extinct cave lion. *Sci. Rep.* **10**, 12621 (2020).

282.	A. J. Stuart, A. M. Lister, Extinction chronology of the cave lion *Panthera spelaea*. *Quat. Sci. Rev.* **30**, 2329–2340 (2011).

283.	M. Pickford, P. Mein, New Pedetidae (Rodentia: Mammalia) from the Mio-Pliocene of Africa. *Estud. Geológicos* **67**, 455–469 (2012).

284.	E. Lukashevich, V. Blagoderov, Review of Mesozoic Perissommatidae (Insecta: Diptera). *Zootaxa* **4718**, 481–496 (2020).

285.	M. Boderau, *et al.*, Phimophorus chiodii sp. nov., first representative of a cryptic assassin bug subfamily from Dominican amber (Reduviidae: Phimophorinae) as revealed with traditional light microscopy and computed micro-tomographic reconstruction. *Palaeoentomology* **7**, 299–305 (2024).

286.	D. Chaverra-Rodríguez, D. Forero, N. Jaramillo-O, O. Triana-Ch, New record and ecological notes of Phimophorus spissicornis (Hemiptera: Heteroptera: Reduviidae: Phimophorinae) in Colombia. *Rev. Colomb. Entomol.* **36**, 176–178 (2010).

287.	H. J. Campbell, J. A. Grant‐Mackie, Jurassic Pholadomyidae (Bivalvia) from New Zealand and New Caledonia. *N. Z. J. Geol. Geophys.* **38**, 47–59 (1995).

288.	J. M. Diaz, J. M. Diaz, F. Gast, D. C. Torres, Rediscovery of a Caribbean living fossil: Pholadomya candida GB Sowerby I, 1823 (Bivalvia: Anomalodesmata: Pholadomyoidea). *The Nautilus* **123**, 19--20 (2009).

289.	D. G. Lazo, The bivalve Pholadomya gigantea in the Early Cretaceous of Argentina:: Taxonomy, taphonomy, and paleogeographic implications. *Acta Palaeontol. Pol.* **52**, 375–390 (2007).

290.	M. Zakhera, M. El-Hedeny, A. El-Sabbagh, S. Al Farraj, Callovian–Oxfordian bivalves from central Saudi Arabia: Systematic paleontology and paleobiogeography. *J. Afr. Earth Sci.* **130**, 60–75 (2017).

291.	P. Sztajner, The mode of life in the genus Pholadomya as inferred from the fossil record. *Geosciences* **10**, 397 (2020).

292.	C. E. Terhune, T. Gaudin, S. Curran, A. Petculescu, The youngest pangolin (Mammalia, Pholidota) from Europe. *J. Vertebr. Paleontol.* **41**, e1990075 (2021).

293.	D. M. Alba, A. S. Hammond, V. Vinuesa, I. Casanovas-Vilar, First record of a Miocene pangolin (Pholidota, Manoidea) from the Iberian Peninsula. *J. Vertebr. Paleontol.* **38**, e1424716 (2018).

294.	J.-Y. Crochet, L. Hautier, T. Lehmann, A pangolin (Manidae, Pholidota, Mammalia) from the French Quercy phosphorites (Pech du Fraysse, Saint-Projet, Tarn-et-Garonne, late Oligocene, MP 28). *Palaeovertebrata* **39**, e4 (2015).

295.	W.-L. Fu, *et al.*, A new species of Protamphisopus Nicholls (Crustacea, Isopoda, Phreatoicidea) from Middle Triassic Luoping Fauna of Yunnan Province, China. *J. Paleontol.* **84**, 1003–1013 (2010).

296.	F. R. Schram, Isopod from the Pennsylvanian of Illinois. *Science* **169**, 854–855 (1970).

297.	G. D. F. Wilson, S. Shaik, Y. Ranga Reddy, A new species of Andhracoides Wilson and Ranga Reddy, 2011 (Isopoda: Hypsimetopidae) from Belum Cave, Andhra Pradesh, India, with a phylogenetic review of the family. *J. Crustac. Biol.* **35**, 216–240 (2015).

298.	G. D. F. Wilson, Y. R. Reddy, Andhracoides shabuddin gen. nov., sp. nov., a new phreatoicidean isopod (Crustacea, Hypsimetopidae) from hypogean aquatic habitats in Andhra Pradesh, India. *Zootaxa* **2869**, 37–53 (2011).

299.	S. Wedmann, S. Bradler, J. Rust, The first fossil leaf insect: 47 million years of specialized cryptic morphology and behavior. *Proc. Natl. Acad. Sci. U. S. A.* **104**, 565–569 (2007).

300.	G. W. Stull, F. Herrera, S. R. Manchester, C. Jaramillo, B. H. Tiffney, Fruits of an “Old World” tribe (Phytocreneae; Icacinaceae) from the Paleogene of North and South America. *Syst. Bot.* **37**, 784–794 (2012).

301.	G. W. Stull, R. Duno de Stefano, D. E. Soltis, P. S. Soltis, Resolving basal lamiid phylogeny and the circumscription of Icacinaceae with a plastome-scale data set. *Am. J. Bot.* **102**, 1794–1813 (2015).

302.	G. Mayr, The Paleogene fossil record of birds in Europe. *Biol. Rev. Camb. Philos. Soc.* **80**, 515–542 (2005).

303.	R. A. Pyron, F. T. Burbrink, J. J. Wiens, A phylogeny and revised classification of Squamata, including 4161 species of lizards and snakes. *BMC Evol. Biol.* **13**, 93 (2013).

304.	C. Perrin, F. R. Bosellini, Paleobiogeography of scleractinian reef corals: Changing patterns during the Oligocene–Miocene climatic transition in the Mediterranean. *Earth-Sci. Rev.* **111**, 1–24 (2012).

305.	A. Vertino, J. Stolarski, F. R. Bosellini, M. Taviani, “Mediterranean Corals Through Time: From Miocene to Present” in *The Mediterranean Sea: Its History and Present Challenges*, S. Goffredo, Z. Dubinsky, Eds. (Springer Netherlands, 2014), pp. 257–274.

306.	C. Perrin, W. Kiessling, “Latitudinal Trends in Cenozoic Reef Patterns and their Relationship to Climate” in *Carbonate Systems during the Oligocene–Miocene Climatic Transition*, (John Wiley & Sons, Ltd, 2012), pp. 17–33.

307.	A. P. Selvatti, A. Galvão, G. Mayr, C. Y. Miyaki, C. A. de M. Russo, Southern hemisphere tectonics in the Cenozoic shaped the pantropical distribution of parrots and passerines. *J. Biogeogr.* **49**, 1753–1766 (2022).

308.	D. T. Ksepka, J. A. Clarke, A new stem parrot from the Green River Formation and the complex evolution of the grasping foot in Pan-Psittaciformes. *J. Vertebr. Paleontol.* **32**, 395–406 (2012).

309.	D. M. Waterhouse, B. E. K. Lindow, N. V. Zelenkov, G. J. Dyke, Two New Parrots (psittaciformes) from the Lower Eocene Fur Formation of Denmark. *Palaeontology* **51**, 575–582 (2008).

310.	G. Mayr, A. C. Kitchener, The Vastanavidae and Messelasturidae (Aves) from the early Eocene London Clay of Walton-on-the-Naze (Essex, UK). *Neues Jahrb. Für Geol. Paläontol. - Abh.* 113–139 (2023). https://doi.org/10.1127/njgpa/2023/1119.

311.	G. Mayr, Mousebirds (Coliiformes), parrots (Psittaciformes), and other small birds from the late Oligocene/early Miocene of the Mainz Basin, Germany. *Neues Jahrb. Für Geol. Paläontol. - Abh.* 129–144 (2010). https://doi.org/10.1127/0077-7749/2010/0089.

312.	G. J. Dyke, J. H. Cooper, A new psittaciform bird from the London Clay (Lower Eocene) of England. *Palaeontology* **43**, 271–285 (2000).

313.	H. Yan, *et al.*, Biogeographic history of Pterocarya (Juglandaceae) inferred from phylogenomic and fossil data. *J. Syst. Evol.* **n/a**.

314.	Q. Zhang, R. H. Ree, N. Salamin, Y. Xing, D. Silvestro, Fossil-Informed Models Reveal a Boreotropical Origin and Divergent Evolutionary Trajectories in the Walnut Family (Juglandaceae). *Syst. Biol.* **71**, 242–258 (2022).

315.	E. Barrón, *et al.*, “The Fossil History of Quercus” in *Oaks Physiological Ecology. Exploring the Functional Diversity of Genus Quercus L.*, E. Gil-Pelegrín, J. J. Peguero-Pina, D. Sancho-Knapik, Eds. (Springer International Publishing, 2017), pp. 39–105.

316.	E. B. Leopold, G. Liu, A long pollen sequence of Neogene age, Alaska range. *Quat. Int.* **22–23**, 103–140 (1994).

317.	F. Grímsson, G. W. Grimm, R. Zetter, T. Denk, Cretaceous and Paleogene Fagaceae from North America and Greenland: evidence for a Late Cretaceous split between Fagus and the remaining Fagaceae. *Acta Palaeobot.* **56**, 247–305 (2016).

318.	F. Grímsson, *et al.*, Fagaceae pollen from the early Cenozoic of West Greenland: revisiting Engler’s and Chaney’s Arcto-Tertiary hypotheses. *Plant Syst. Evol.* **301**, 809–832 (2015).

319.	J. A. Wolfe, T. Tanai, “The Miocene Seldovia Point flora from the Kenai Group, Alaska” (U.S. Govt. Print. Off., 1980).

320.	D. A. Fordham, *et al.*, 52,000 years of woolly rhinoceros population dynamics reveal extinction mechanisms. *Proc. Natl. Acad. Sci.* **121**, e2316419121 (2024).

321.	D. Fraser, N. Rybczynski, M. Gilbert, M. R. Dawson, Mid-Cenozoic rhinocerotid dispersal via the North Atlantic. *Nat. Ecol. Evol.* **9**, 2223–2237 (2025).

322.	R. H. L. Disney, The scuttle fly genus Rhopica Schmitz (Diptera: Phoridae). *Ann. Zool.* **52**, 285–291 (2002).

323.	C. Meloro, M. E. H. Jones, Tooth and cranial disparity in the fossil relatives of Sphenodon (Rhynchocephalia) dispute the persistent ‘living fossil’ label. *J. Evol. Biol.* **25**, 2194–2209 (2012).

324.	J. M. Waters, D. Craw, Goodbye Gondwana? New Zealand Biogeography, Geology, and the Problem of Circularity. *Syst. Biol.* **55**, 351–356 (2006).

325.	S. Anantharaman, *et al.*, First rhynchocephalian (Reptilia, Lepidosauria) from the Cretaceous–Paleogene of India. *J. Vertebr. Paleontol.* **42**, e2118059 (2022).

326.	S. Apesteguía, R. O. Gómez, G. W. Rougier, The youngest South American rhynchocephalian, a survivor of the K/Pg extinction. *Proc. R. Soc. B Biol. Sci.* **281**, 20140811 (2014).

327.	S. Apesteguía, M. E. H. Jones, A Late Cretaceous “tuatara” (Lepidosauria: Sphenodontinae) from South America. *Cretac. Res.* **34**, 154–160 (2012).

328.	S. Apesteguía, G. W. Rougier, A late Campanian sphernodontid maxilla from northern Patagonia. *Am. Mus. Novit.* **2007**, 1–11 (2007).

329.	S. E. Evans, D. Sigogneau-Russell, New sphenodontians (Diapsida: Lepidosauria: Rhynchocephalia) from the Early Cretaceous of North Africa. *J. Vertebr. Paleontol.* **17**, 45–51 (1997).

330.	V. H. Reynoso, An unusual aquatic sphenodontian (Reptilia: Diapsida) from the Tlayua Formation (Albian), Central Mexico. *J. Paleontol.* **74**, 133–148 (2000).

331.	M. E. H. Jones, A. J. D. Tennyson, J. P. Worthy, S. E. Evans, T. H. Worthy, A sphenodontine (Rhynchocephalia) from the Miocene of New Zealand and palaeobiogeography of the tuatara (Sphenodon). *Proc. R. Soc. B Biol. Sci.* **276**, 1385–1390 (2009).

332.	E.-M. Sadowski, *et al.*, Carnivorous leaves from Baltic amber. *Proc. Natl. Acad. Sci.* **112**, 190–195 (2015).

333.	T. Yang, *et al.*, Boreotropical range expansion and long-distance dispersal explain two amphi-Pacific tropical disjunctions in Sabiaceae. *Mol. Phylogenet. Evol.* **124**, 181–191 (2018).

334.	J. A. Wolfe, “Paleogene floras from the Gulf of Alaska region.” (U.S. Govt. Print. Off., 1977).

335.	C. Mourer-Chauviré, J. Cheneval, Les Sagittariidae fossiles (Aves, Accipitriformes) de l’Oligocène des phosphorites du Quercy et du Miocène inférieur de Saint-Gérand-le-Puy. *Geobios* **16**, 443–459 (1983).

336.	M. Mills, D. Schreve, O. Middleton, C. J. Sandom, Going back for the future: Incorporating Pleistocene fossil records of saiga antelope into habitat suitability models. *J. Biogeogr.* **51**, 1351–1364 (2024).

337.	J. Jürgensen, *et al.*, Diet and habitat of the saiga antelope during the late Quaternary using stable carbon and nitrogen isotope ratios. *Quat. Sci. Rev.* **160**, 150–161 (2017).

338.	P. (Philip) Allsworth-Jones, *The Szeletian and the transition from Middle to Upper Paleolithic in central Europe /* (Clarendon Press ; Oxford University Press, 1986).

339.	A. A. Andreev, *et al.*, Weichselian and Holocene palaeoenvironmental history of the Bol’shoy Lyakhovsky Island, New Siberian Archipelago, Arctic Siberia. *Boreas* **38**, 72–110 (2009).

340.	P. F. Campos, *et al.*, Ancient DNA sequences point to a large loss of mitochondrial genetic diversity in the saiga antelope (Saiga tatarica) since the Pleistocene. *Mol. Ecol.* **19**, 4863–4875 (2010).

341.	S. Collcutt, A. Currant, C. Hawkes, A further report on the excavations at Sun Hole, Cheddar. *Proc. Univ. Bristol Spelaeol. Soc.* **16**, 21–38 (1981).

342.	A. Currant, Late Pleistocene saiga antelope Saiga tatarica on Mendip. *Proc. Univ. Bristol Spelaeol. Soc.* **18**, 74–80 (1987).

343.	D. Jurestovsky, T. A. Joyner, Applications of species distribution modeling for palaeontological fossil detection: late Pleistocene models of Saiga (Artiodactyla: Bovidae, Saiga tatarica). *Palaeobiodiversity Palaeoenvironments* **98**, 277–285 (2018).

344.	P. Kosintsev, Late Pleistocene large mammal faunas from the Urals. *Quat. Int.* **160**, 112–120 (2007).

345.	F. B. Lanoë, S. Péan, A. Yanevich, Saiga antelope hunting in Crimea at the Pleistocene–Holocene transition: the site of Buran-Kaya III Layer 4. *J. Archaeol. Sci.* **54**, 270–278 (2015).

346.	E. Crégut-Bonnoure, S. Gagnière, Sur la présence de Saiga tatarica (Mammalia, Artiodactyla) dans le dépôt pléistocène supérieur de la grotte de La Salpêtrière à Remoulins (Gard, France). *Nouv. Archéologiques Muséed’Histoire Nat. Lyon* **19**, 37–42 (1981).

347.	A. Nadachowski, G. Lipecki, U. Ratajczak, K. Stefaniak, P. Wojtal, Dispersal events of the saiga antelope (Saiga tatarica) in Central Europe in response to the climatic fluctuations in MIS 2 and the early part of MIS 1. *Quat. Int.* **420**, 357–362 (2016).

348.	B. Ridush, *et al.*, Emine-Bair-Khosar Cave in the Crimea, a huge bone accumulation of Late Pleistocene fauna. *Quat. Int.* **284**, 151–160 (2013).

349.	E. Sidorchuk, M. Bertrand, New fossil labidostomatids (Acari: Labidostomatidae) from Eocene amber and presence of an apustulate species in Europe. *Acarologia* **53**, 25–39 (2013).

350.	A. Bannikov, J. Tyler, C. Sorbini, Two new taxa of Eocene rabbitfi shes (Perciformes, Siganidae) from the North Caucasus (Russia), with redescription of Acanthopygaeus agassizi (Eastman) from Monte Bolca (Italy) and a phylogenetic analysis of the family. *Boll. Mus. Civ. Storia Nat. Verona Geol. Paleontol. Preistoria* **34**, 3–21 (2010).

351.	D. R. Bellwood, C. H. R. Goatley, O. Bellwood, The evolution of fishes and corals on reefs: form, function and interdependence. *Biol. Rev.* **92**, 878–901 (2017).

352.	A. C. Siqueira, D. R. Bellwood, P. F. Cowman, Historical biogeography of herbivorous coral reef fishes: The formation of an Atlantic fauna. *J. Biogeogr.* **46**, 1611–1624 (2019).

353.	S. L. Olson, An Early Eocene Oilbird from the Green River Formation of Wyoming (Caprimulgiformes : Steatornithidae). (1987).

354.	L. Janz, R. G. Elston, G. S. Burr, Dating North Asian surface assemblages with ostrich eggshell: implications for palaeoecology and extirpation. *J. Archaeol. Sci.* **36**, 1982–1989 (2009).

355.	N. V. Zelenkov, A. V. Lavrov, D. B. Startsev, I. A. Vislobokova, A. V. Lopatin, A giant early Pleistocene bird from eastern Europe: unexpected component of terrestrial faunas at the time of early Homo arrival. *J. Vertebr. Paleontol.* **39**, e1605521 (2019).

356.	L. Barton, P. J. Brantingham, D. Ji, “Late Pleistocene climate change and Paleolithic cultural evolution in northern China: Implications from the Last Glacial Maximum” in *Developments in Quaternary Sciences*, Late Quaternary Climate Change and Human Adaptation in Arid China., (Elsevier, 2007), pp. 105–128.

357.	C. Jouault, B. Coutret, K. O. Konhauser, A. Nel, New odonatans (Odonata: Gomphaeschnidae; Synlestidae) from the Paleocene Paskapoo Formation: systematic and biogeographical implications. *J. Syst. Palaeontol.* **21**, 2261457 (2023).

358.	A. P. Rasnitsyn, S. I. Golovatch, The identity of phryssonotus burmiticus (Cockerell, 1917) (Diplopoda, Polyxenida, synxenidae) in cretaceous amber from Myanmar. *J. Syst. Palaeontol.* **2**, 153–157 (2004).

359.	K. Vohland, M. Hamer, A review of the millipedes (Diplopoda) of Namibia, with identification keys and descriptions of two new genera and five new species. *Afr. Invertebr.* **54**, 251–304 (2013).

360.	R. W. Graham, F. Grady, T. M. Ryan, Juvenile Pleistocene tapir skull from Russells Reserve Cave, Bath County, Virginia: Implications for cold climate adaptations. *Quat. Int.* **530–531**, 35–41 (2019).

361.	J. S. Zijlstra, L. J. Flynn, W. Wessels, The westernmost tarsier: A new genus and species from the Miocene of Pakistan. *J. Hum. Evol.* **65**, 544–550 (2013).

362.	Z. Kvaček, S. R. Manchester, H. E. Schorn, Cones, seeds, and foliage of Tetraclinis salicornioides (Cupressaceae) from the Oligocene and Miocene of western North America: A geographic extension of the European Tertiary species. *Int. J. Plant Sci.* **161**, 331–344 (2000).

363.	Y. Fu, C. Cai, D. Huang, First hairy cicadas in mid-Cretaceous amber from northern Myanmar (Hemiptera: Cicadoidea: Tettigarctidae). *Cretac. Res.* **93**, 285–291 (2019).

364.	H. Jiang, *et al.*, Mesozoic evolution of cicadas and their origins of vocalization and root feeding. *Nat. Commun.* **15**, 376 (2024).

365.	U. Kaulfuss, M. Moulds, A new genus and species of tettigarctid cicada from the early Miocene of New Zealand: Paratettigarcta zealandica (Hemiptera, Auchenorrhyncha, Tettigarctidae). *ZooKeys* 83–94 (2015). https://doi.org/10.3897/zookeys.484.8883.

366.	B. Wang, H. Zhang, Tettigarctidae (Insecta: Hemiptera: Cicadoidea) from the Middle Jurassic of Inner Mongolia, China. *Geobios* **42**, 243–253 (2009).

367.	Y. Yan, *et al.*, Phytogeographic history of the tea family Inferred through high-resolution phylogeny and fossils. *Syst. Biol.* **70**, 1256–1271 (2021).

368.	Y. Na, J. Blanchard, H. Wang, Fruits, seeds and flowers from the Puryear clay pit (middle Eocene Cockfield Formation), western Tennessee, USA. *Palaeontol. Electron.* **23**, 1–57 (2020).

369.	THE ANGIOSPERM PHYLOGENY GROUP, An update of the Angiosperm Phylogeny Group classification for the orders and families of flowering plants: APG III. *Bot. J. Linn. Soc.* **161**, 105–121 (2009).

370.	R. López-Antoñanzas, S. Sen, P. Mein, Systematics and phylogeny of the cane rats (Rodentia: Thryonomyidae). *Zool. J. Linn. Soc.* **142**, 423–444 (2004).

371.	L. J. Flynn, *et al.*, “Siwalik Rodent Assemblages for NOW: Biostratigraphic Resolution in the Neogene of South Asia” in *Evolution of Cenozoic Land Mammal Faunas and Ecosystems: 25 Years of the NOW Database of Fossil Mammals*, I. Casanovas-Vilar, L. W. van den Hoek Ostende, C. M. Janis, J. Saarinen, Eds. (Springer International Publishing, 2023), pp. 43–58.

372.	L. J. Flynn, Small mammal indicators of forest paleoenvironment in the Siwalik deposits of the Potwar Plateau, Pakistan. *Deinsea* **10**, 183–196 (2003).

373.	L. J. Flynn, A. J. Winkler, Dispersalist implications of Paraulacodus indicus: A South Asian rodent of African affinity. *Hist. Biol.* **9**, 223–235 (1994).

374.	S. Chen, *et al.*, The earliest Timematids in Burmese amber reveal diverse tarsal pads of stick insects in the mid-Cretaceous. *Insect Sci.* **26**, 945–957 (2019).

375.	L. D. Martin, Paleogene avifauna of the Holarctic. *Vertebr. Palasiat.* **48**, 367 (2010).

376.	J. A. Guzmán-Sandoval, G. E. Rössner, Miocene chevrotains (Mammalia, Artiodactyla, Tragulidae) from Pakistan. *Hist. Biol.* **33**, 743–776 (2021).

377.	B. Mennecart, *et al.*, The first French tragulid skull (Mammalia, Ruminantia, Tragulidae) and associated tragulid remains from the Middle Miocene of Contres (Loir-et-Cher, France). *Comptes Rendus Palevol* **17**, 189–200 (2018).

378.	S. Roussiakis, “The fossil record of tragulids (Mammalia: Artiodactyla: Tragulidae) in Greece” in *Fossil Vertebrates of Greece Vol. 2: Laurasiatherians, Artiodactyles, Perissodactyles, Carnivorans, and Island Endemics*, E. Vlachos, Ed. (Springer International Publishing, 2022), pp. 335–350.

379.	S. Schneider, F. T. Fürsich, W. Werner, Biometric methods for species recognition in Trigonia Bruguière (Bivalvia; Trigoniidae): a case study from the Upper Jurassic of Western Europe. *Paläontol. Z.* **85**, 257–267 (2011).

380.	Z. Bochenski, Z. M. Bochenski, An Old World hummingbird from the Oligocene: a new fossil from Polish Carpathians. *J. Ornithol.* **149**, 211–216 (2008).

381.	G. Mayr, Old World Fossil Record of Modern-Type Hummingbirds. *Science* **304**, 861–864 (2004).

382.	G. Mayr, New trogons from the early Tertiary of Germany. *Ibis* **147**, 512–518 (2005).

383.	G. Mayr, A well-preserved second trogon skeleton (Aves, Trogonidae) from the middle Eocene of Messel, Germany. *Palaeobiodiversity Palaeoenvironments* **89**, 1–6 (2009).

384.	G. Mayr, V. L. De Pietri, A. C. Kitchener, Narrow-beaked trogons from the early Eocene London Clay of Walton-on-the-Naze (Essex, UK). *J. Ornithol.* **164**, 749–764 (2023).

385.	R. G. Moyle, Phylogeny and biogeographical history of Trogoniformes, a pantropical bird order. *Biol. J. Linn. Soc.* **84**, 725–738 (2005).

386.	A. V. Kristoffersen, An early Paleogene trogon (Aves: Trogoniformes) from the Fur Formation, Denmark. *J. Vertebr. Paleontol.* **22**, 661–666 (2002).

387.	T. Lehmann, Phylogeny and systematics of the Orycteropodidae (Mammalia, Tubulidentata). *Zool. J. Linn. Soc.* **155**, 649–702 (2009).

388.	S. Roussiakis, N. Kargopoulos, P. Kampouridis, G. Svorligkou, G. Theodorou, The fossil aardvark Amphiorycteropus gaudryi (Forsyth Major, 1888) from the late Miocene of Kerassia (Euboea, Greece). *Hist. Biol.* **34**, 493–506 (2022).

389.	N. Spassov, T. Tzankov, D. Geraads, Late Neogene stratigraphy, biochronology, faunal diversity and environments of South-West Bulgaria (Struma River Valley). *Geodiversitas* **28** (2006).

390.	C. I. Forsyth Major, Exhibition of, and remarks upon, the tooth of an ant-bear (Orycteropus) from the Upper Miocene of Maragha (Persia). *Proc. Zool. Soc. Lond.* 239–240 (1893).

391.	D. A. G. López, P. E. Ortiz, M. C. M. Jaén, M. S. Moyano, First Record of Arctotherium (Ursidae, Tremarctinae) in Northwestern Argentina and its Paleobiogeographic Significance. *J. Vertebr. Paleontol.* **28**, 1232–1237 (2008).

392.	L. H. Soibelzon, E. P. Tonni, M. Bond, The fossil record of South American short-faced bears (Ursidae, Tremarctinae). *J. South Am. Earth Sci.* **20**, 105–113 (2005).

393.	D. D. Stynder, L. R. G. DeSantis, S. L. Donohue, B. W. Schubert, P. S. Ungar, A Dental Microwear Texture Analysis of the Early Pliocene African Ursid Agriotherium africanum (Mammalia, Carnivora, Ursidae). *J. Mamm. Evol.* **26**, 505–515 (2019).

394.	L. Dong, *et al.*, A new stem-varanid lizard (Reptilia, Squamata) from the early Eocene of China. *Philos. Trans. R. Soc. B Biol. Sci.* **377**, 20210041 (2022).

395.	T. Denk, *et al.*, Cenozoic migration of a desert plant lineage across the North Atlantic. *New Phytol.* **238**, 2668–2684 (2023).

396.	M. B. Lara, A. P. Rasnitsyn, A. M. Zavattieri, Potrerilloxyela menendezi gen. et sp. nov. from the Late Triassic of Argentina: The oldest representative of Xyelidae (Hymenoptera: Symphyta) for Americas. *Paleontol. J.* **48**, 182–190 (2014).

397.	T. Schlüter, Moltenia rieki n. gen., n. sp. (Hymenoptera: Xyelidae?, a tentative sawfly from the Molteno Formation (Upper Triassic), South Africa. *Paläontol. Z.* **74**, 75–78 (2000).

398.	A. J. Maile, W. L. Smith, M. P. Davis, A total-evidence phylogenetic approach to understanding the evolution, depth transitions, and body-shape changes in the anglerfishes and allies (Acanthuriformes: Lophioidei). *PLOS One* **20**, e0322369 (2025).

399.	G. H. F. Azevedo, *et al.*, Fossils constrain biogeographical history in a clade of flattened spiders with transcontinental distribution. *J. Biogeogr.* **48**, 3032–3046 (2021).

400.	N. P. Barker, P. H. Weston, F. Rutschmann, H. Sauquet, Molecular Dating of the “Gondwanan” Plant Family Proteaceae Is Only Partially Congruent with the Timing of the Break-up of Gondwana. *J. Biogeogr.* **34**, 2012–2027 (2007).

401.	C. V. Bennett, P. Upchurch, F. J. Goin, A. Goswami, Deep time diversity of metatherian mammals: implications for evolutionary history and fossil-record quality. *Paleobiology* **44**, 171–198 (2018).

402.	K. Bremer, Ancestral Areas: A Cladistic Reinterpretation of the Center of Origin Concept. *Syst. Biol.* **41**, 436–445 (1992).

403.	J. W. Brown, The discovery of Megalota in the Neotropics, with a revision of the New World species (Lepidoptera: Tortricidae: Olethreutini). *Zootaxa* **2279**, 1–50 (2009).

404.	C. D. Brownstein, A juvenile bird with possible crown-group affinities from a dinosaur-rich Cretaceous ecosystem in North America. *BMC Ecol. Evol.* **24**, 20 (2024).

405.	F. A. Carbonaro, M. C. Langer, S. S. Nihei, G. de Souza Ferreira, R. P. Ghilardi, Inferring ancestral range reconstruction based on trilobite records: a study-case on Metacryphaeus (Phacopida, Calmoniidae). *Sci. Rep.* **8**, 15179 (2018).

406.	J.-C. Castel, M.-P. Coumont, M. Boudadi-Maligne, A. Prucca, Rôle et origine des grands carnivores dans les accumulations naturelles: le cas des loups (Canis lupus) de l’Igue du Gral (Sauliac-sur-Célé, Lot, France). *Rev. Paléobiol.* **29**, 411–425 (2010).

407.	A. A. Chiarenza, *et al.*, 100 million years of turtle paleoniche dynamics enable the prediction of latitudinal range shifts in a warming world. *Curr. Biol.* **33**, 109-121.e3 (2023).

408.	R. L. Cifelli, Chapter 5: Marsupial Mammals from the Albian–Cenomanian (Early–Late Cretaceous) Boundary, Utah. *Bull. Am. Mus. Nat. Hist.* **2004**, 62–79 (2004).

409.	R. L. Cifelli, B. M. Davis, B. Sames, Earliest Cretaceous mammals from the western United States. *Acta Palaeontol. Pol.* **59**, 31–52 (2012).

410.	S. Claramunt, J. Cracraft, A new time tree reveals Earth history’s imprint on the evolution of modern birds. *Sci. Adv.* **1**, e1501005 (2015).

411.	L. G. Cook, M. D. Crisp, Not so ancient: the extant crown group of Nothofagus represents a post-Gondwanan radiation. *Proc. R. Soc. B Biol. Sci.* **272**, 2535–2544 (2005).

412.	Z. Csiki-Sava, E. Buffetaut, A. Ősi, X. Pereda-Suberbiola, S. L. Brusatte, Island life in the Cretaceous - faunal composition, biogeography, evolution, and extinction of land-living vertebrates on the Late Cretaceous European archipelago. *ZooKeys* 1–161 (2015). https://doi.org/10.3897/zookeys.469.8439.

413.	J. J. F. E. de Wilde, M. Hughes, M. Rodda, D. C. Thomas, Pliocene intercontinental dispersal from Africa to Southeast Asia highlighted by the new species Begonia afromigrata (Begoniaceae). *Taxon* **60**, 1685–1692 (2011).

414.	A. Derevianko, S. Gladyshev, T. Nohrina, J. Olsen, The Mongolian Early Holocene excavations at Chikhen Agui rockshelter in the Gobi Altai. *Rev. Archaeol.* **24**, 50–56 (2003).

415.	D. G. Drucker, D. Henry-Gambier, Determination of the dietary habits of a Magdalenian woman from Saint-Germain-la-Rivière in southwestern France using stable isotopes. *J. Hum. Evol.* **49**, 19–35 (2005).

416.	K. Yu. Eskov, E. D. Lukashevich, On the history of ranges of two relict nematoceran families, Ptychopteridae and Tanyderidae (Insecta: Diptera): a biogeographical puzzle. *rej* **24**, 257–270 (2015).

417.	R. M. Feldmann, C. E. Schweitzer, Paleobiogeography of Southern Hemisphere decapod Crustacea. *J. Paleontol.* **80**, 83–103 (2006).

418.	D. J. Field, J. Benito, A. Chen, J. W. M. Jagt, D. T. Ksepka, Late Cretaceous neornithine from Europe illuminates the origins of crown birds. *Nature* **579**, 397–401 (2020).

419.	J. A. Finarelli, J. J. Flynn, Ancestral State Reconstruction of Body Size in the Caniformia (Carnivora, Mammalia): The Effects of Incorporating Data from the Fossil Record. *Syst. Biol.* **55**, 301–313 (2006).

420.	G. Fiorentino, *et al.*, Deep time extinction of largest insular ant predators and the first fossil Neoponera (Formicidae: Ponerinae) from Miocene age Dominican amber. *BMC Biol.* **21**, 26 (2023).

421.	D. G. Gavin, *et al.*, Climate refugia: joint inference from fossil records, species distribution models and phylogeography. *New Phytol.* **204**, 37–54 (2014).

422.	G. Giribet, *et al.*, The ‘Peripatos’ in Eurogondwana? – Lack of evidence that south-east Asian onychophorans walked through Europe. *Invertebr. Syst.* **32**, 842–865 (2018).

423.	P. Grandcolas, R. Nattier, S. Trewick, Relict species: a relict concept? *Trends Ecol. Evol.* **29**, 655–663 (2014).

424.	D. Grimaldi, M. S. Engel, Extralimital Fossils of the “Gondwanan” Family Sphaeropsocidae (Insecta: Psocodea). *Am. Mus. Novit.* **2006**, 1–18 (2006).

425.	M. G. Harasewych, “Pleurotomarioidean gastropods” in *Advances in Marine Biology*, Molluscan Radiation - Lesser-known Branches., (Academic Press, 2002), pp. 237–294.

426.	Q. He, J. R. Prado, L. L. Knowles, Inferring the geographic origin of a range expansion: Latitudinal and longitudinal coordinates inferred from genomic data in an ABC framework with the program x-origin. *Mol. Ecol.* **26**, 6908–6920 (2017).

427.	C. P. Heesy, N. J. Stevens, K. E. Samonds, “Biogeographic origins of primate higher taxa” in *Primate Biogeography: Progress and Prospects*, S. M. Lehman, J. G. Fleagle, Eds. (Springer US, 2006), pp. 419–437.

428.	A. H. Jahren, The Arctic forest of the Middle Eocene. *Annu. Rev. Earth Planet. Sci.* **35**, 509–540 (2007).

429.	Y. Jiang, *et al.*, The importance of the North Atlantic land bridges and eastern Asia in the post-Boreotropical biogeography of the Northern Hemisphere as revealed from the poison ivy genus (*Toxicodendron*, Anacardiaceae). *Mol. Phylogenet. Evol.* **139**, 106561 (2019).

430.	S. Ladevèze, C. Selva, C. de Muizon, What are “opossum-like” fossils? The phylogeny of herpetotheriid and peradectid metatherians, based on new features from the petrosal anatomy. *J. Syst. Palaeontol.* **18**, 1463–1479 (2020).

431.	B. B. Lamont, T. He, R. M. Cowling, Fossil pollen resolves origin of the South African Proteaceae as transcontinental not transoceanic. *Ann. Bot.* **133**, 649–658 (2024).

432.	E. G. Leigh, A. O’Dea, G. J. Vermeij, Historical biogeography of the Isthmus of Panama. *Biol. Rev.* **89**, 148–172 (2014).

433.	H. Li, Early Cretaceous sarraceniacean-like pitcher plants from China. *Acta Bot. Gallica* **152**, 227–234 (2005).

434.	Y.-D. Li, E. Tihelka, A. F. Newton, D.-Y. Huang, C.-Y. Cai, New species of Notocupes (Coleoptera: Archostemata) from the Middle Jurassic Daohugou beds, with discussion on the generic circumscription. *Palaeoentomology* **6**, 398–415 (2023).

435.	B. S. Lieberman, Phylogenetic biogeography with and without the fossil record: gauging the effects of extinction and paleontological incompleteness. *Palaeogeogr. Palaeoclimatol. Palaeoecol.* **178**, 39–52 (2002).

436.	B. S. Lieberman, Paleobiogeography: The Relevance of Fossils to Biogeography. *Annu. Rev. Ecol. Evol. Syst.* **34**, 51–69 (2003).

437.	J. G. Lundberg, J. P. Sullivan, R. Rodiles-Hernández, D. A. Hendrickson, Discovery of African roots for the Mesoamerican Chiapas catfish, Lacantunia enigmatica, requires an ancient intercontinental passage. *Proc. Acad. Nat. Sci. Phila.* **156**, 39–53 (2007).

438.	Z.-X. Luo, Q. Ji, J. R. Wible, C.-X. Yuan, An Early Cretaceous tribosphenic mammal and metatherian evolution. *Science* **302**, 1934–1940 (2003).

439.	S. R. Manchester, B. H. Tiffney, Integration of paleobotanical and neobotanical data in the assessment of phytogeographic history of Holarctic angiosperm clades. *Int. J. Plant Sci.* **162**, S19–S27 (2001).

440.	G. Mayr, Avian higher level biogeography: Southern Hemispheric origins or Southern Hemispheric relicts? *J. Biogeogr.* **44**, 956–958 (2017).

441.	A. S. Meseguer, F. L. Condamine, Ancient tropical extinctions at high latitudes contributed to the latitudinal diversity gradient*. *Evolution* **74**, 1966–1987 (2020).

442.	C. Mourer-Chauviré, Les oiseaux fossiles des phosphorites du quercy (éocène supérieur a oligocène supérieur): Implications paléobiogéographiques. *Geobios* **15**, 413–426 (1982).

443.	Z.-L. Nie, *et al.*, Climate-influenced boreotropical survival and rampant introgressions explain the thriving of New World grapes in the north temperate zone. *J. Integr. Plant Biol.* **65**, 1183–1203 (2023).

444.	L. R. Parenti, M. C. Ebach, Evidence and hypothesis in biogeography. *J. Biogeogr.* **40**, 813–820 (2013).

445.	P. T. Pennington, Q. C. B. Cronk, J. A. Richardson, R. J. Burnham, K. R. Johnson, South American palaeobotany and the origins of neotropical rainforests. *Philos. Trans. R. Soc. Lond. B. Biol. Sci.* **359**, 1595–1610 (2004).

446.	M. D. Pirie, G. Litsios, D. U. Bellstedt, N. Salamin, J. Kissling, Back to Gondwanaland: can ancient vicariance explain (some) Indian Ocean disjunct plant distributions? *Biol. Lett.* **11**, 20150086 (2015).

447.	P. Posadas, J. V. Crisci, L. Katinas, Historical biogeography: A review of its basic concepts and critical issues. *J. Arid Environ.* **66**, 389–403 (2006).

448.	Ş. Procheş, S. Ramdhani, Eighty-three lineages that took over the world: a first review of terrestrial cosmopolitan tetrapods. *J. Biogeogr.* **40**, 1819–1831 (2013).

449.	Ş. Procheş, S. Ramdhani, S. J. Perera, J. R. Ali, S. Gairola, Global hotspots in the present-day distribution of ancient animal and plant lineages. *Sci. Rep.* **5**, 15457 (2015).

450.	T. B. Quental, C. R. Marshall, Diversity dynamics: molecular phylogenies need the fossil record. *Trends Ecol. Evol.* **25**, 434–441 (2010).

451.	C. J. Raxworthy, M. R. J. Forstner, R. A. Nussbaum, Chameleon radiation by oceanic dispersal. *Nature* **415**, 784–787 (2002).

452.	R. H. Ree, S. A. Smith, Maximum Likelihood Inference of Geographic Range Evolution by Dispersal, Local Extinction, and Cladogenesis. *Syst. Biol.* **57**, 4–14 (2008).

453.	S. Richter, M. Schwentner, C. S. Wirkner, S. T. Ahyong, Phylogeny and species diversity of Tasmanian mountain shrimps and their relatives (Crustacea, Anaspidesidae). *Zool. Scr.* **47**, 84–105 (2018).

454.	E. F. Riek, Fossil insects from the Triassic beds at Mt. Crosby, Queensland. *Aust. J. Zool.* **3**, 654–691 (1955).

455.	F. Ronquist, Ancestral Areas and Parsimony. *Syst. Biol.* **43**, 267–274 (1994).

456.	B. Roth, K. C. Emberton, “Extralimital” Land Mollusks (Gastropoda) from the Deep River Formation, Montana: Evidence for Mesic Medial Tertiary Climate. *Proc. Acad. Nat. Sci. Phila.* **145**, 93–106 (1994).

457.	M. Sanz-Arnal, C. Benítez-Benítez, M. Miguez, P. Jiménez-Mejías, S. Martín-Bravo, Are Cenozoic relict species also climatic relicts? Insights from the macroecological evolution of the giant sedges of Carex sect. Rhynchocystis (Cyperaceae). *Am. J. Bot.* **109**, 115–129 (2022).

458.	E. E. Saupe, *et al.*, Climatic shifts drove major contractions in avian latitudinal distributions throughout the Cenozoic. *Proc. Natl. Acad. Sci.* **116**, 12895–12900 (2019).

459.	E. E. Saupe, *et al.*, Spatio-temporal climate change contributes to latitudinal diversity gradients. *Nat. Ecol. Evol.* **3**, 1419–1429 (2019).

460.	S. Scarpetta, The earliest known occurrence of Elgaria (Squamata: Anguidae) and a minimum age for crown Gerrhonotinae: Fossils from the Split Rock Formation, Wyoming, USA. *Palaeontol. Electron.* **21**, 1–8 (2018).

461.	A. Shukla, R. C. Mehrotra, P. Verma, K. Chandra, A. Singh, “Out-of-India” dispersal for Adina (tribe Naucleeae; family Rubiaceae): evidence from the early Eocene fossil record from India. *Palaeoworld* **30**, 737–745 (2021).

462.	H. Singh, Palaeoenvironmental and taphonomic biases in palynological assemblages preserved in amber versus sediments from the Umarsar Lignite, Kutch Basin, Gujarat, India. *Hist. Biol.* **33**, 2305–2315 (2021).

463.	K. T. Smith, *et al.*, The biodiversity of the Eocene Messel Pit. *Palaeobiodiversity Palaeoenvironments* **104**, 859–940 (2024).

464.	T. Smith, J. Habersetzer, N. B. Simmons, G. F. Gunnell, “Systematics and paleobiogeography of early bats” in *Evolutionary History of Bats: Fossils, Molecules and Morphology*, Cambridge Studies in Morphology and Molecules: New Paradigms in Evolutionary Bio., G. F. Gunnell, N. B. Simmons, Eds. (Cambridge University Press, 2012), pp. 23–66.

465.	N. Stanczak, *et al.*, A new pseudoscorpion genus (Garypinoidea: Garypinidae) from the Eocene supports extinction and range contraction in the European paleobiota. *PeerJ* **11**, e15989 (2023).

466.	T. A. Stidham, D. Lofgren, A. A. Farke, M. Paik, R. Choi, A lithornithid (Aves: Palaeognathae) from the Paleocene (Tiffanian) of southern California. *PaleoBios* **31** (2014).

467.	J. Szwedo, “The traps of the ‘amber trap’. Amber-trapped insects trap scientists with enigmas.” in (2009), pp. 155–169.

468.	V. da C. Tavares, O. M. Warsi, F. Balseiro, C. A. Mancina, L. M. Dávalos, Out of the Antilles: Fossil phylogenies support reverse colonization of bats to South America. *J. Biogeogr.* **45**, 859–873 (2018).

469.	A. H. Thornhill, S. Y. W. Ho, C. Külheim, M. D. Crisp, Interpreting the modern distribution of Myrtaceae using a dated molecular phylogeny. *Mol. Phylogenet. Evol.* **93**, 29–43 (2015).

470.	P. Upchurch, Gondwanan break-up: legacies of a lost world? *Trends Ecol. Evol.* **23**, 229–236 (2008).

471.	M. Wang, Z. Zhou, “The evolution of birds with implications from new fossil evidences” in *The Biology of the Avian Respiratory System: Evolution, Development, Structure and Function*, J. N. Maina, Ed. (Springer International Publishing, 2017), pp. 1–26.

472.	K. Widrig, D. J. Field, The evolution and fossil record of palaeognathous birds (Neornithes: Palaeognathae). (2022). https://doi.org/10.3390/d14020105.

473.	T. E. Williamson, S. L. Brusatte, T. D. Carr, A. Weil, B. R. Standhardt, The phylogeny and evolution of Cretaceous–Palaeogene metatherians: cladistic analysis and description of new early Palaeocene specimens from the Nacimiento Formation, New Mexico. *J. Syst. Palaeontol.* **10**, 625–651 (2012).

474.	T. E. Williamson, S. L. Brusatte, G. P. Wilson, The origin and early evolution of metatherian mammals: the Cretaceous record. *ZooKeys* 1–76 (2014). https://doi.org/10.3897/zookeys.465.8178.

475.	W. O. Wong, D. L. Dilcher, C. C. Labandeira, G. Sun, A. Fleischmann, Early Cretaceous Archaeamphora is not a carnivorous angiosperm. *Front. Plant Sci.* **6** (2015).

476.	G. C. Young, Analytical methods in palaeobiogeography, and the role of early vertebrate studies. *Palaeoworld* **19**, 160–173 (2010).

477.	G. C. Young, C. J. Burrow, J. A. Long, S. Turner, B. Choo, Devonian macrovertebrate assemblages and biogeography of East Gondwana (Australasia, Antarctica). *Palaeoworld* **19**, 55–74 (2010).

478.	T. Yu, T. A. Neubauer, A. Jochum, First freshwater gastropod preserved in amber suggests long-distance dispersal during the Cretaceous Period. *Geol. Mag.* **158**, 1327–1334 (2021).
